# Protein–Protein Interaction Stabilizers from MD Simulation-Derived Pharmacophores

**DOI:** 10.1021/acs.jcim.6c00290

**Published:** 2026-05-01

**Authors:** Mohd Ibrahim, Martin Zacharias

**Affiliations:** Physics Department and Center for Functional Protein Assemblies, 9184 Technical University of Munich, 85748 Garching, Germany

## Abstract

Protein–protein interactions (PPI) play a crucial role in nearly all cellular processes, and their dysregulation often leads to diseases. Stabilizing rather than inhibiting PPIs by small drug-like molecules offers a promising route to modulate PPIs. Here, we present an effective workflow (PPIS–MDPharma) to identify PPI stabilizers solely from molecular dynamic (MD) simulation trajectories of protein–protein (PP) complexes in the absence of a stabilizer and large database pharmacophore screening. Our approach involves extracting pharmacophore features, namely, hydrogen bonding, electrostatic, hydrophobic, and aromatic features from MD simulation by analyzing the interaction of the interface pocket residues with water and ions. The resulting pharmacophore model, along with tens of thousands of derived subsets, is ranked and screened against a local database of 50 million compounds using rapid pharmacophore screening. It yields tens of thousands of stabilizer candidates followed by rescoring using the molecular mechanics generalized Born surface area (MMGBSA) method. For seven PP complexes, the top-ranked ligands exhibited MMGBSA scores similar to experimentally known stabilizers. The approach is computationally more efficient than alternative docking based methods, making it a promising tool for discovering novel PPI stabilizers for various therapeutic applications.

## Graphical abstract

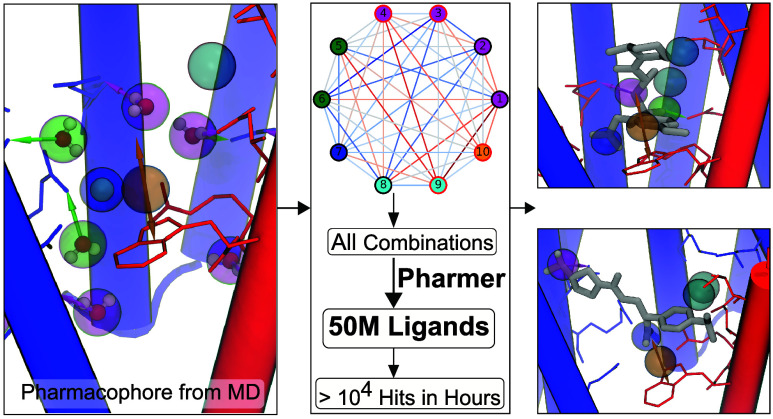

## Introduction

Protein–protein interactions (PPI) play central roles in virtually all cellular processes, including signal transduction, structural assembly, metabolic regulation, and immune responses.^1–3^ PPIs are involved in numerous human diseases, making them attractive targets for therapeutic modulation using small molecules.^4,5^ Most studies have focused on inhibition based PPI modulation^4,5^ however, stabilization of PPI via small molecules offers a promising alternative^6–8^ since both binding and dissociation of PP complexes are biologically important. Small molecule stabilizers can enhance or restore functional complexes offering new opportunities for diseases where protein assemblies are weakened or transiently disrupted.^6,9,10^ For example, the ubiquitin–proteasome system (UPS), a central regulator of protein degradation, has been modulated through small-molecule stabilization of the transient interaction between ubiquitin and the E2 enzyme Cdc34A, with important implications for diseases such as cancer.^11,12^ Stabilization of the MDM2 protein dimer with small molecules blocks MDM2 binding to p53, thereby preserving p53’s tumor suppressor activity^13^ and stabilization of adapter protein 14–3–3 with various binding partners modulates cellular activities like signaling and trafficking.^14–16^ Despite these examples, the discovery of small–molecule PPI stabilizers has largely relied on target-specific experimental studies, and systematic computational exploration of such designs remains far less developed than inhibitor–based approaches. A recent study have proposed a docking–based computational workflow to identify PPI stabilizers *in silico*.^9^ However, such approaches are constrained by the substantial computational cost of docking, which limits screening to only a few million compounds within a reasonable period of time, and limitations due to the often poor performance of docking scoring functions.^17^

Pharmacophore–based methods provide an efficient alternative concept for identifying small–molecule binders and enable rapid screening of ultralarge libraries containing hundreds of millions of compounds,^18–20^ and in some benchmark studies have been shown to achieve higher hit rates than docking-based virtual screening.^21^ However, pharmacophore models are most often derived from static ligand conformations or single protein–ligand complexes,^19,22^ or directly from protein structures using known binding pockets.^23^ As a result, these approaches frequently fail to capture protein and ligand flexibility and generally neglect solvent-mediated interactions that can be critical for molecular recognition.^24,25^

All-atom molecular dynamics (MD) simulations overcome key limitations of static models by explicitly capturing conformational flexibility, solvent effects, and interactions at atomic resolution.^24–26^ Analysis of water and ion interactions in binding-site regions has enabled the derivation of pharmacophore features and has been successfully applied to single-protein systems.^27,28^ However, existing approaches exhibit notable limitations: some generate highly complex pharmacophores with more than hundred features, requiring manual selection of the most relevant interactions, and may fail when the protein deviates from the ligand–bound conformation during simulations.^27^ Other approaches depend on carefully tuned parameters for specific systems to create a unique pharmacophore model limiting transferability.^28^ All rely on commercial software fully or in the screening phase and have mostly been evaluated in retrospective studies. Moreover, current MD–based pharmacophore methods focus exclusively on single protein chains. In contrast, orthosteric PPI stabilizers require simultaneous interactions to both protein partners. A recent MD simulation study of 18 PP complexes with known stabilizers showed that effective stabilizers typically act via a dual-binding mechanism, exhibiting comparable interactions toward both proteins.^9^ Explicit consideration of this dual–binding requirement is therefore essential for stabilizer screening. Analysis of 226 PP complexes in the same study found that >75% have druggable interface cavities suitable for binding small drug-like molecules. Such cavities can be detected by various pocket detection algorithms like Fpocket,^29^ DeepPocket^30^ or related methods. Pharmacophore features can be extracted by analyzing the interaction between the pocket residues/atoms and water and ions.

In the current study, we present an effective method for identifying PPI stabilizers by combining MD–derived pharmacophores with large compound database screening in an automated way. Applying our protocol to seven important PP complexes, we identified the top 10 stabilizer candidates with estimated MMGBSA binding affinities that are comparable to, and in several cases more favorable, than known stabilizers. To facilitate broader use, we have made our workflow publicly available together with a database of 10 million compounds randomly selected from the ZINC20 database (like the 50 million used in this study) at https://github.com/ibrahim-mohd/PPIS-MDPharma, allowing for rapid implementation and use.

## Methods

### All-Atom Simulations

All atom simulations of various PP complexes were performed using the Gromacs package (v-2023 and v-2025).^31^ The complexes were selected from a subset of an earlier related study by Chen and Zacharias.^9^ Each PP complex was placed in a dodecahedron box with a size such that the minimum distance between protein and edge of the box is 1.2 nm and were solvated with the 4-site OPC water model.^32^ Sodium and Chloride ions were added to neutralize the systems and to achieve a salt concentration of 150 mM. The systems were energy minimized using the gradient descent method followed by equilibration MD runs done in two steps (i) the backbone atoms of the protein were restrained with force constants 10^3^, 10^2^, 10.0, 1.0 kJ mol^–1^ nm^–2^. For each force constant the simulations were run for 200 ps. (ii) 1.0 ns long unrestrained equilibration MD runs were performed using the C-rescale barostat^33^ with time constant of 5.0 ps. Subsequently, production runs were carried out for 100 ns with the pressure maintained at 1.0 bar using the Parinello-Rahman isotropic barostat^34^ with a time constant of 5.0 ps. Temperature was set to 300 K using the stochastic velocity rescaling thermostat^35^ with a time constant of 1.0 ps. Electrostatic interactions were evaluated using the particle mesh Ewald method^36^ with a real space cutoff at 1.2 nm. Van-der-Waals interactions were truncated and shifted to zero at 1.2 nm. All bonds involving hydrogen were constrained using the LINCS method.^37^ The Newtons equations of motion were integrated using a leapfrog integrator with a time step of 2.0 fs.

Water was described using the OPC model,^32^ ions with Joung and Cheatham model^38^ and proteins with the ff19SB force field.^39^ For the simulations in the presence of ligands, ff14SB force field^40^ was used for protein with the TIP3P^41^ water model and the GAFF2 force field^42^ was used for the ligands. The protonation state of ligands were predicted at pH 7.0 using OpenBabel.^43^ The partial charges for ligands were assigned according to the AM1-BCC method^44^ using Antechamber, part of AmberTools,^45^ and GROMACS topology files were obtained using the acpype.py script.^46^ All analysis was performed with scripts using MDAnalysis^47^ or with Gromacs tools and VMD was used for all visualizations.^48^ Neutral capping groups NME and ACE were added using the script at https://github.com/ibrahim-mohd.

### MMGBSA Free Energy Calculations

Interaction free energies between the ligands and protein complexes were estimated using the molecular mechanics generalized Born/surface area (MMGBSA) method,^49^ as implemented in MMPBSA.py^50^ within the AmberTools23 environment.^45^ An internal dielectric constant of 1 and an external dielectric constant of 80 were applied, and effective Born radii were computed using the GBOBCII model (igb = 5).^51^ For each protein–ligand system, three independent 10.0 ns simulations were performed using Gromacs 2025. The resulting trajectories were converted to the corresponding Amber formats using MDAnalysis^47^ and ParmEd for compatibility with MMPBSA.py. For each trajectory, 70 frames were selected for MMGBSA analysis after discarding the first 2 ns for equilibration.

### Local Ligand Database

We used the open-source software Pharmer^20^ to search for ligands that matched specified pharmacophores. For efficient searching, Pharmer requires ligand databases in a binary format, which can be generated from .sdf files using the tool itself. We obtained a total of 300 million ligands in .sdf format from the ZINC20 database,^52^ from which 50 million ligands were randomly selected and converted into Pharmer-compatible local databases using Pharmer. Because Pharmer does not support very large single databases, the 50 million ligands were divided into 10 separate databases, each containing approximately 5 million ligands. The complete database set occupied approximately 700 GB of disk space.

## Results

### Pharmacophore Features from All-Atom MD Simulations

An overview of our workflow for deriving pharmacophores from all-atom MD simulations of PP complexes is shown in Figure 1. Starting from the apo or ligand free holo form of a PP complex, we perform all-atom MD simulations in explicit water at a specified ion concentration (150 mM) for 100 ns. Following the simulations, the ligand binding pocket at the PP interface is identified using the pocket-detection tool Fpocket,^29^ or, when a ligand is known, extracted directly from the PP–ligand complex. Residues located within 5 Å of the pocket probes (in the case of Fpocket) or of the ligand are considered pocket residues. These residues are then analyzed to identify key pharmacophore features, including (i) hydrogen–bond donor and acceptor sites, (ii) hydrophobic and aromatic sites, and (iii) positively and negatively charged (Coulombic interaction) sites.

**Figure 1:**
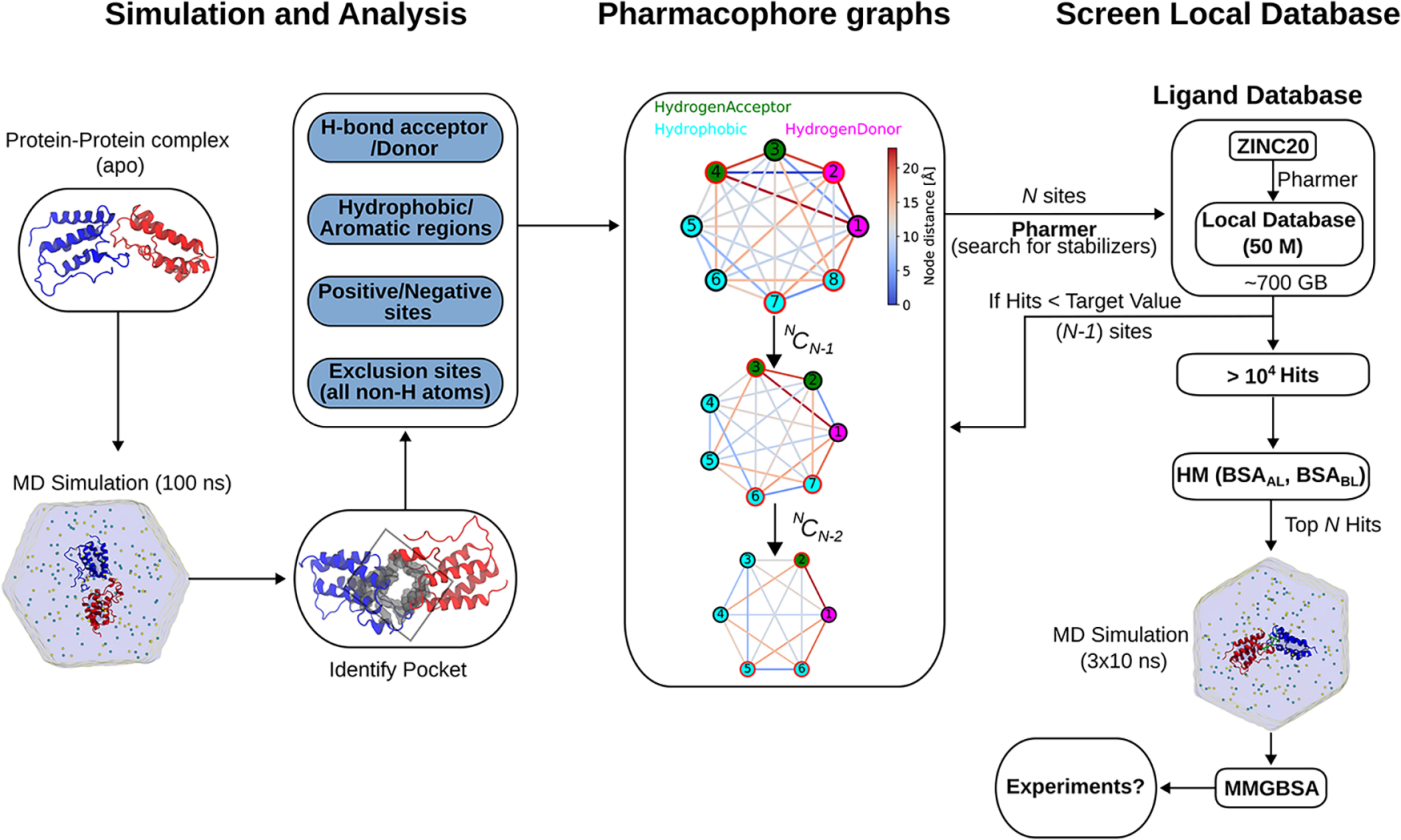
Overview of workflow. First, the PP complex is simulated in explicit water with ions and analyzed to extract relevant pharmacophore features. A master pharmacophore model is then constructed based on the entire MD simulation trajectory, and all possible subsets are generated by iteratively removing one feature at a time. These pharmacophore models are subsequently used to screen a local compound database. The resulting hits are filtered based on the buried surface area (BSA) that the compounds share with each protein partner to identify dual binding candidates. The selected candidates are further evaluated using the MMGBSA approach selecting compounds with similar mean interaction to both protein partners.

#### Hydrophobic and Aromatic Interaction Sites

The hydrophobic and aromatic interaction sites are obtained by calculating the solvation free energy (Δ*G*_sol_) of each pocket residue, a positive value of which indicates a hydrophobic or an aromatic residue (Figure 2A). The solvation free energy for each residue is obtained as sum of individual atom contributions multiplied by the respective solvent accessible surface area (SASA)^53^ which is implemented with the Gromacs gmx sasa routine. Hydrophobic interaction sites involving phenylalanine, tyrosine, or tryptophan residues are classified as aromatic sites.

**Figure 2:**
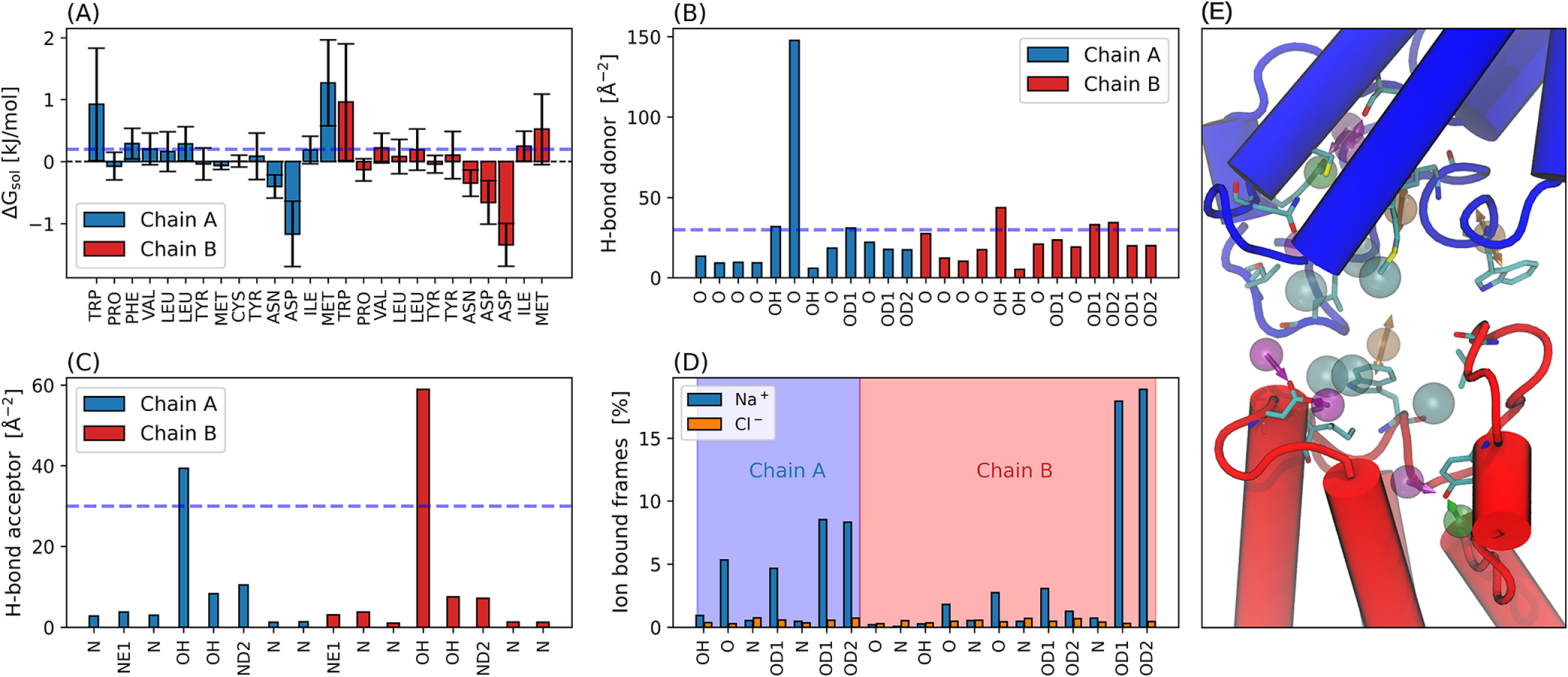
Pharmacophore features from MD simulation of the bromodomain dimer (5ad3). (A) Solvation free energy for each pocket residue, computed as the time average over the entire simulation trajectory using gmx sasa function in Gromacs. Error bars represent the standard deviation over the trajectory. (B) SASA normed H-bond donor and (C) H-bond acceptor frequency (D) Cation (Na^+^) and anion (Cl^–^) accumulation sites. The bars show the percentage of MD frames in which an anion or cation comes in contact with a given atom in the pocket. A contact is defined when distance between ion and the pocket atoms is <4 Å. Such feature plots for all the other studied systems are shown in the SI (Figures S1–S6). (E) Pharmacophore features generated from the sites identified in panels (A–D). The transparent spheres represents the pharmacophore features: hydrophobic (cyan), aromatic (orange), H-bond donor (magenta) and H-bond acceptor (green). Arrows indicate the directions of hydrogen-bond and aromatic features according to the Pharmer convention.

#### Hydrogen Bond Donor/Acceptor

To obtain hydrogen bond donor and acceptor sites, all the oxygen and nitrogen atoms of the pocket residues are analyzed for hydrogen bonding with water molecules using the inbuilt function in MDAnalysis^47^ with a distance and angle cutoff of 3 Å and 150° respectively. The obtained H-bond count is further normalized by the solvent-accessible surface area (SASA) of the respective atoms, since more exposed atoms otherwise invariably exhibit a higher H-bond count. For example, the resulting normed H-bond donor and acceptor frequencies for the bromodomain dimer (PDB: 5ad3) pocket atoms are shown in Figure 2 B,C respectively.

#### Electrostatic Interaction Sites

To obtain the positive and negative electrostatic interaction sites the ionic atmosphere for each atom in the pocket is analyzed. A pocket atom is assumed to be in contact with an ion if the distance between them is less 4 Å. For each potential ion binding site, the contact with both cations (Na^+^) and anions (Cl^–^) is counted and the percentage of simulation frames in which an ion is bound to the site is displayed (Figure 2D).

#### Exclusion Sites

In pharmacophore search exclusion site refer to the region in the pocket not accessible to the ligands. Here, we render all the non-hydrogen atoms of the protein structure as exclusion sites, this is a highly stringent criterion and ensures that only ligands that fit precisely into the pocket are considered.

### Pharmacophore Models as Graphs

For each of the interaction features analyzed above, we applied specific cut-offs to identify sites with high interaction propensity. Across all systems studied, we established the following cutoff ranges: for hydrophobic and aromatic interactions, Δ*G*_sol_ > 0.2 kJ/mol; for hydrogen-bond acceptors or donors, values above 30 yield reliable results. Electrostatic interaction sites are determined by applying the cutoff to the difference between positive and negative ion-bound percentages, with a threshold of 25.0%. For instance, in Figure 2, using a cutoff of 30 for hydrogen-bond donors and acceptors, 0.2 kJ/mol for hydrophobic and aromatic sites, and 25% for positive/negative sites, we identify 6 hydrogen-bond donors, 2 hydrogen-bond acceptors, 6 hydrophobic sites, and 3 aromatic sites, resulting in a total of 17 interaction sites. Together, these 17 sites define the pharmacophore model, which is characterized by two main features: (i) the interaction type, i.e., hydrogen-bond donor, hydrogen-bond acceptor, aromatic, hydrophobic, positive, negative, or exclusion site, and (ii) the position of each site in Cartesian coordinates. For hydrogen-bond and aromatic interactions, an additional directional feature is included, represented by a unit vector. To enable efficient manipulation, these features are represented using a graph data structure (Figure 1), as implemented in the NetworkX Python package.^54^ The pharmacophore model containing all potential sites is termed the *master pharmacophore*, from which subsets are derived by taking various possible combinations.

To determine the pharmacophore feature positions we first performed a clustering analysis based on the RMSD of the interface residues using the gmx cluster function in Gromacs. The centroid (cluster center) of the dominant cluster was then chosen as the representative structure for the pharmacophore search.

#### Pharmacophore Feature Position

The three-dimensional coordinates of pharmacophore features are assigned using the MD simulation frame selected for the pharmacophore search. The positions of hydrogen-bond donor and acceptor features are defined by the instantaneous positions of water oxygen atoms that form hydrogen bonds with the identified sites in the selected frame, using distance and angle cutoffs of 4.0 Å and 150°, respectively. In cases where no water molecule satisfies these criteria, the angle cutoff is iteratively reduced to a minimum of 100°. The hydrogen-bond direction is defined by the unit vector connecting the donor and acceptor atoms in accordance with the Pharmer convention.

Positive and negative feature positions are given by the location of bound positive or negative ions within a distance cutoff varied from 2.0 to 5.0 Å. If no ion is detected within this range, the position is assigned using the same procedure as for hydrophobic features described below.

For hydrophobic features, the positions are obtained by translating the coordinate of the identified site (the residue center of mass in the case of hydrophobic sites) by 4.0 Å along the vector pointing from the site toward the geometric center of the binding pocket. For aromatic features, the position is obtained by translating the geometric center of the identified residue by 3.0 Å along the vector normal to the aromatic ring plane. Since two opposite normal vectors are possible, the one that places the pharmacophore feature closer to the pocket center is selected. The direction of aromatic feature is given by the two opposite unit vectors normal to the ring plane in accordance with Pharmer convention.

#### Pharmacophore Score

The master pharmacophore graph obtained above is converted into a Pharmer-compatible JSON file. Using this file as input, a pharmacophore search is performed with the Pharmer software on a locally created database to identify ligands that satisfy the defined pharmacophore. In most cases, no or too few (<10) ligands fully satisfy the master pharmacophore; therefore, subsets of the master pharmacophore are generated by systematically considering all possible combinations of features. For a master pharmacophore with *N* features (excluding the volume exclusion features), the next subset corresponds to combinations of $\left(\begin{array}{l} N\\ N-1 \end{array}\right)$ sites, and if no match is found, the process continues with $\left(\begin{array}{l} N\\ N-2 \end{array}\right)$ sites, and so on (Figure 1). For a master pharmacophore containing more than 10 sites, the number of possible subsets grows rapidly as smaller subsets are considered. Therefore, for each subset pharmacophore model, a ranking method is required so that only a percentage of the highest-ranked members of a subsets are considered in subsequent ligand screening or for rescoring later with MMGBSA.

Different pharmacophore features are scored using distinct metrics–hydrophobic and aromatic sites by solvation free energy, hydrogen bonds by SASA normalized frequency, and electrostatic interactions by fraction of bound ions. To combine these scores, each feature type is first normalized by its respective maximum value; for example, in the top graph of Figure 1, the four hydrophobic interactions are each divided by the highest solvation free energy among them to obtain their normalized scores. The total score of the pharmacophore model is then calculated as the sum of the normalized scores of all constituent sites. To ensure the dual binding mode of stabilization, the total score is further multiplied by a symmetry factor which is simply given by min­(*N*_*A*_, *N*_*B*_)/max­(*N*_*A*_, *N*_*B*_), where *N*_*A*_ and *N*_*B*_ corresponds to the number of pharmacophore sites that comes from each of the protein partner, with *N* = *N*_*A*_ + *N*_*B*_ as the total number of sites. A symmetry value of 1 means equal contribution from both partners, while a value of 0 eliminates cases where only one partner contributes all the sites, thus enforcing dual binding–mode of interactions.

#### Filtering the Pharmacophore Hits

The above procedure yields between 10^4^–10^5^ pharmacophore hits, many of which must be filtered to retain the most promising candidates. For each ligand, we compute the buried surface area (BSA) with each protein partner, denoted as BSA_AL_ and BSA_BL_, using the Shrake-Rupley algorithm^55^ as implemented in the MDTraj Python package.^56^ The ligand score is then calculated as the harmonic mean, HM­(BSA_AL_, BSA_BL_), which in a simple yet effective manner favors ligands sharing similar surface area with each protein partner thus favoring a dual binding-mode of stabilization.

### Application to PP Complexes with Known Stabilizers

We applied stabilizer search approach to seven PP complexes with known small-molecule stabilizers: the Cdc34–ubiquitin 1α dimer (PDB 4mdk^11^); 14–3–3 bound to the plant H^+^-ATPase PMA2 (PDB 3m50 ^16^); BRD4/BRD4 bromodomain dimers (PDB 5ad3 ^57^); 14–3–3/ChREBP (PDB 6ygj^15^) and its apo form (PDB 4gnt); and the apo forms of the immunoglobulin light-chain homodimer (lambda–6A/lambda–6A; PDB 6mg4 ^58^) and iGluR2/iGluR2 (PDB 3b6q^59^). Apo and holo forms correspond to absence or presence of a known stabilizer in the structure, respectively. For the first four complexes, the MD simulations were started from the holo structure after removing the stabilizer, for the remaining systems available apo structures were used. Each complex was simulated for 100 ns, and the experimentally known pocket was used for pharmacophore feature analysis which corresponds to the frequent case of an approximately known or desired stabilizer binding site. Pharmacophore screening details for each system are summarized in Table 1, with feature plots illustrated in Figures S1–S6. All combinations of a master pharmacophore with *N* sites were evaluated; for *N* > 10, screening started from 10–site combinations i.e, $\left(\begin{array}{l} N\\ 10 \end{array}\right)$ and proceeded to smaller subsets, as no ligand satisfies more than 10 features. For subsets with more than 50,000 subpharmacophore models, only the top 50,000 were screened. For most cases, thousands of hits were obtained within a few hours (Table 1), and execution was terminated once hits exceeded 10,000. For the 5ad3 complex, an additional criterion was applied during screening: only pharmacophore models for which the maximum pairwise distance between features exceeded 23 Å were considered, ensuring selection of ligands spanning the full depth of the protein pockets (Figure 2E), which yielded the highest MMGBSA-scoring ligands.

**Table 1:** Overview of Pharmacophore Screening Parameters^a^

	Thresholds				Total Hits							
System	Δ*G*_sol_	Normed Hbond	Anion/Cation	Total Sites	*N* = 8	*N* = 7	*N* = 6	*N* = 5	*N* = 4	Total Hits	Pharmacophore Models Screened	Time (hours)
3m50 (holo)	0.20	30/30	25	9	0	0	4	21246	n.a.	21250	82	0.77
4mdk (holo)	0.20	35/35	25	18	0	0	0	1117	16668	17785	152,436	3.6
5ad3 (holo)	0.20	30/30	25	13	202	17047	n.a	n.a	n.a	17249	4004	3.44
6ygj (holo)	0.20	35/35	25	14	0	1	2996	10880	n.a	13877	12,537	17.19
6mg4 (apo)	0.17	35/35	25	15	0	0	0	31	8079	8110	15,129	3.45
3b6q (apo)	0.17	30/30	25	11	0	0	0	0	15480	15480	1439	0.28
4gnt (apo)	0.20	35/35	25	16	0	55	10158	11916	n.a	22129	21,729	12.70
4gnt ^†^ (apo)	0.20	35/35	25	23	0	1296	8851	n.a	n.a	10147	55,992	26.5

a Four complexes were in the holo form and three in the apo form. The symbol † indicates the case in which no prior information about the binding pocket was assumed; in this case, pockets were first identified using Fpocket.^29^ *N* denotes the number of pharmacophore features included in each combinatorial subset and was varied from 10 to 4 until the maximum number of hits (10^4^) was reached. For 4gnt, searches were additionally performed with *N* = 5 despite the hit limit being reached at *N* = 6. Due to parallel execution, some runs exceeded this limit because the program waits for all active jobs to complete before termination. All screening was performed on a database of 50 million ligands stored on a Solid State Drive (SSD) and using i7 CPUs. Note that the hits are already in a pharmacophore-aligned docked pose, ready for further re-scoring with MD simulation.

The top 50–70 pharmacophore hits with the highest scores, i.e., HM­(BSA_AL_, BSA_BL_), were selected for MD simulations and MMGBSA calculations. For each ligand, MMGBSA interaction free energies with partners A and B (Δ*G*_AL_, Δ*G*_BL_) and the full complex (Δ*G*_(AB)L_) were calculated. The same analysis was applied to experimentally known ligands and compared to the top ten ligands with the lowest Δ*G*_(AB)L_ (see Figure 3).

**Figure 3:**
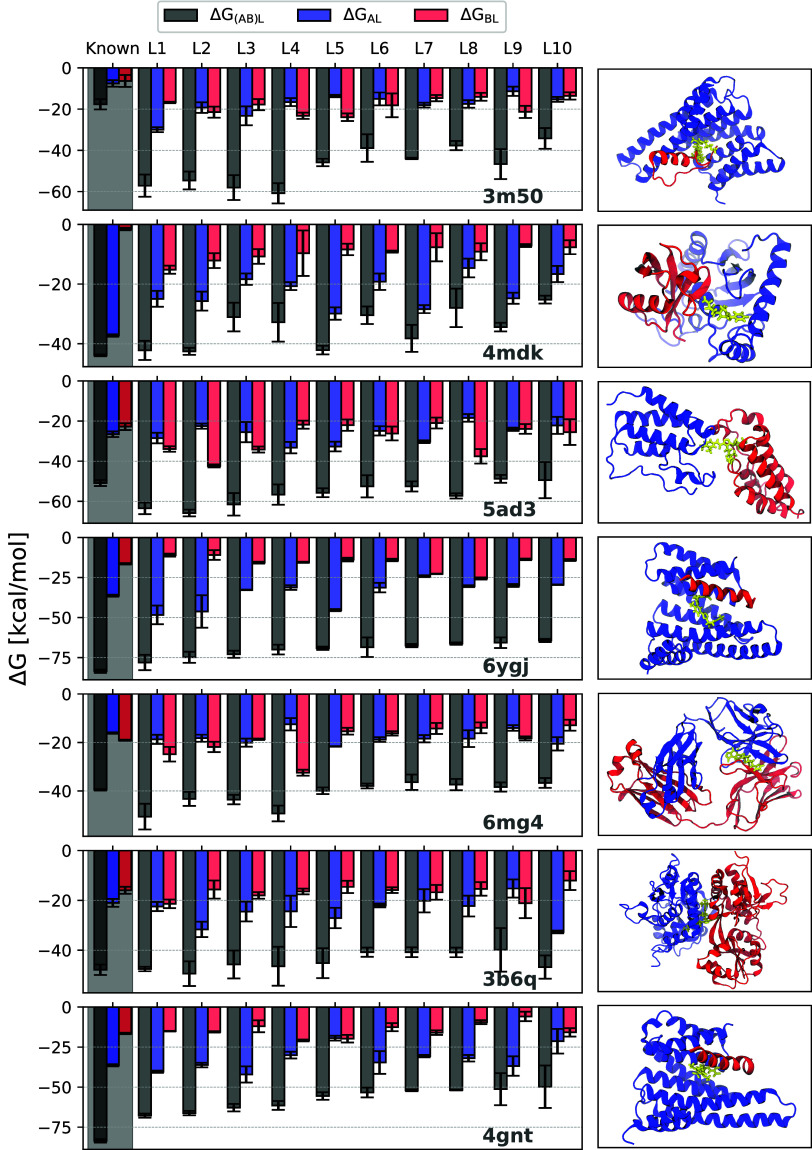
MMGBSA interaction free energies for the top 10 ligands for seven PP complex. For each ligand, the interaction free energies with individual protein chains−Δ*G*_AL_ (light blue) and Δ*G*_BL_ (light red)–as well as the total interaction free energy for the full complex, Δ*G*_(AB)L_ (gray), are shown. For each PP system, the gray shaded region on the left represents the corresponding free energy estimates for the experimentally known stabilizers. The corresponding PP complex structure with one of the top ligands bound are shown on the right. In all these cases, the experimentally known binding pocket was used. The ZINC IDs and chemical structure of the top ligands shown here are presented in the SI (Figures S7–S13).

In the 14–3–3/PMA2 (3m50) complex, the estimated energies for all ten top scoring compounds were significantly more favorable than those of the known ligand (Figure 3). For the other complexes, the estimated free energies of the top pharmacophore hits were generally comparable to those of the known stabilizers. Chemical structures and ZINC IDs of the top ligands are illustrated in the SI (Figures S7–S13, binding poses of top-ranked compounds are shown in Figure 4). In several cases, e.g., 4gnt, the predicted binding pose of the top-ranked compound closely matches that of the known ligand (Figure 4A).

**Figure 4:**
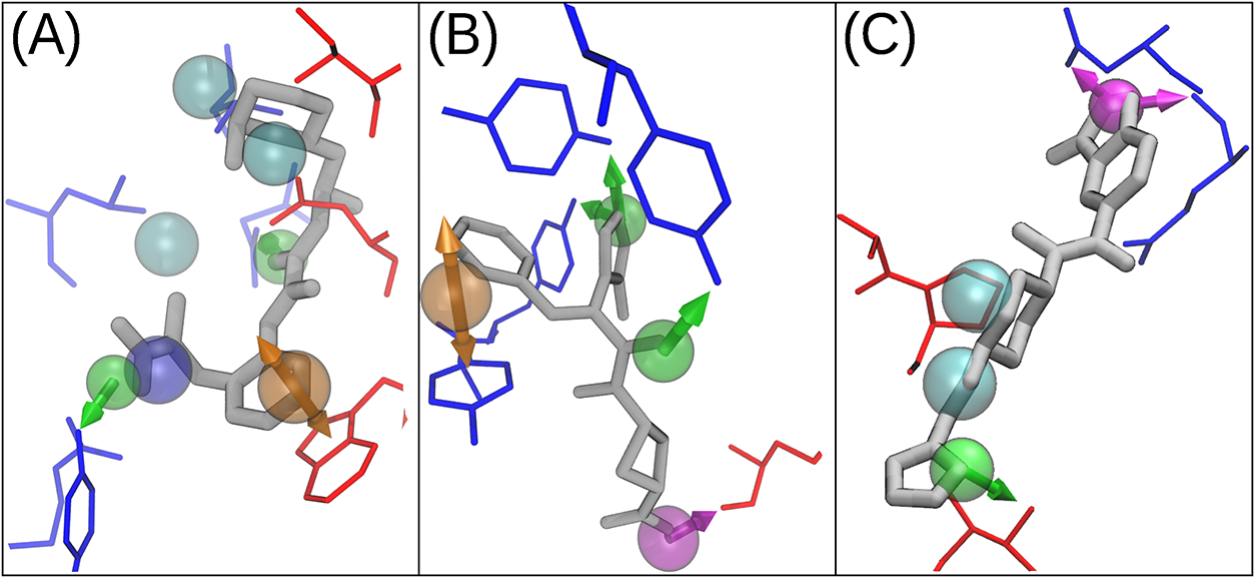
Representative hit poses. Pharmer-generated poses showing pharmacophore hit ligands (gray licorice) within the binding pocket. For clarity only the protein residues responsible for the pharmacophore features are shown in blue and red stick representation. (A) 4gnt, (B) 4mdk, and (C) 6mg4 PP complexes. Transparent spheres indicate pharmacophore features: hydrophobic (cyan), aromatic (orange), negative (blue), hydrogen-bond donor (magenta), and hydrogen-bond acceptor (green). Arrows indicate the directions of hydrogen-bond and aromatic features according to the Pharmer convention.

#### Alternative Scoring and Filtering with Boltz-2

We rescored the MMGBSA-scored ligands for all systems using Boltz-2,^60^ a machine-learning–based method that predicts PP–ligand complex structures from protein sequences and ligand SMILES strings and also estimates ligand binding affinity and binder probability. For each system, we predicted the PP–ligand complex to evaluate whether Boltz-2 places the ligand in the same binding pocket as our protocol, how the predicted binding affinity and binder probability compare with MMGBSA score trends, and whether the predicted complex structures are similar to experimental structures. For almost all systems, Boltz-2 placed the majority of predicted stabilizer ligands (>60%) in the same pocket as the pharmacophore hits, as judged by a pocket residue overlap greater than 50% (SI Figure S14A–S20A). An exception was 3b6q and 3m50, where only ∼5% and ∼18% of ligands showed such overlap (SI Figure S16A, Figure S20A). However, performing the same analysis using AlphaFold-3 (AF3)^61^ resulted in ∼83% and ∼36% of ligands exhibiting pocket residue overlap >50% for 3b6q and 3m50, respectively, while showing Boltz-2–like overlap fractions for all other systems (SI Figure S21). Overall, the percentage of pharmacophore hits with a combined binding-site residue overlap >50% with Boltz-2 or AF3 predictions was 100%, 93%, 92%, 89%, 86%, 81%, and 54% for 5ad3, 4mdk, 6mg4, 4gnt, 3b6q, 6ygj, and 3m50, respectively (SI Figure S21). Notably, no significant correlation was observed between AF3- and Boltz-2-predicted ligand binding-sites for most cases (SI Figure S21). For the binding affinities, in all cases, at most a weak correlation was observed between Boltz-2–predicted affinities and MMGBSA interaction free energies of the stabilizer bound complexes (Δ*G*_(AB)L_) (SI Figures S14B–S20B). Encouragingly, Boltz-2 predicted higher affinities for most pharmacophore hits than for experimentally known ligands, except for 5ad3 and 3b6q, where the known stabilizers resulted in highest predicted affinities relative to nearly all hits (SI Figures S14B–S20B). However, the predicted Boltz-2 binder probabilities are lower for most hits compared to the known ligands, except for the 3m50 ligand, which both Boltz-2 and MMGBSA ranked poorly across all metrics (Figure 3, top; Figures S14C–S20C).

Finally, we filtered the pharmacophore hits for the 5ad3 and 6ygj complexes using Boltz-2 predicted binding affinity and binder probability instead of buried surface area. Simulations of ligands with high Boltz-2 scores resulted in overall less favorable MMGBSA-scoring ligands compared to preselection using BSA (SI Figures S24–S27). Nevertheless, the total MMGBSA binding free energy (Δ*G*_(AB)L_) was < −30 kcal/mol for the majority of cases, which is still very favorable, and a good balance for the interaction with both protein partners was observed in most cases. Hence, compound selection with Boltz-2 offers an alternative to prescoring using BSA but it is more time-consuming (screening of 1000 ligands per complex takes a day using an RTX-4090 GPU workstation).

#### Finding Stabilizers without Prior Pocket Information

For the 14–3–3/ChREBP complex we applied the pharmacophore search approach using the apo complex (without stabilizer, PDB 4gnt) and without assuming any prior knowledge of the stabilizer binding region. Pockets were identified using Fpocket^29^ on 500 simulation frames. Treating pocket probes as ligands, we calculated the buried surface area (BSA) of each pocket with the two protein partners for each frame, and selected the frame with the largest value of HM­(BSA_AL_, BSA_BL_), the harmonic mean of the ligand’s BSA with both protein partners, for pharmacophore screening. Residues within 5.0 Å of the pocket probes were then analyzed for pharmacophore features, as described previously. This approach yielded 30 pharmacophore features, compared to 16 when knowledge of the stabilizer binding site was included (see Table 1). The MMGBSA scores of the top-ranked ligands were comparable to those obtained using known pocket information (SI Figure S30). The mode of interaction of the top ranked stabilizer candidates is also similar to the experimentally known stabilizer (illustrated in Figure 5). However, the larger number of features (30 versus 16, SI Figures S29 and S5) resulted in over 50,000 pharmacophore models being screened, as opposed to around 20,000 when knowledge of the binding region is included, starting from models with seven features or less. The workflow in case of no prior knowledge of the stabilizer binding region is fully integrated in our method requiring only the trajectory XTC file and TPR as inputs.

**Figure 5:**
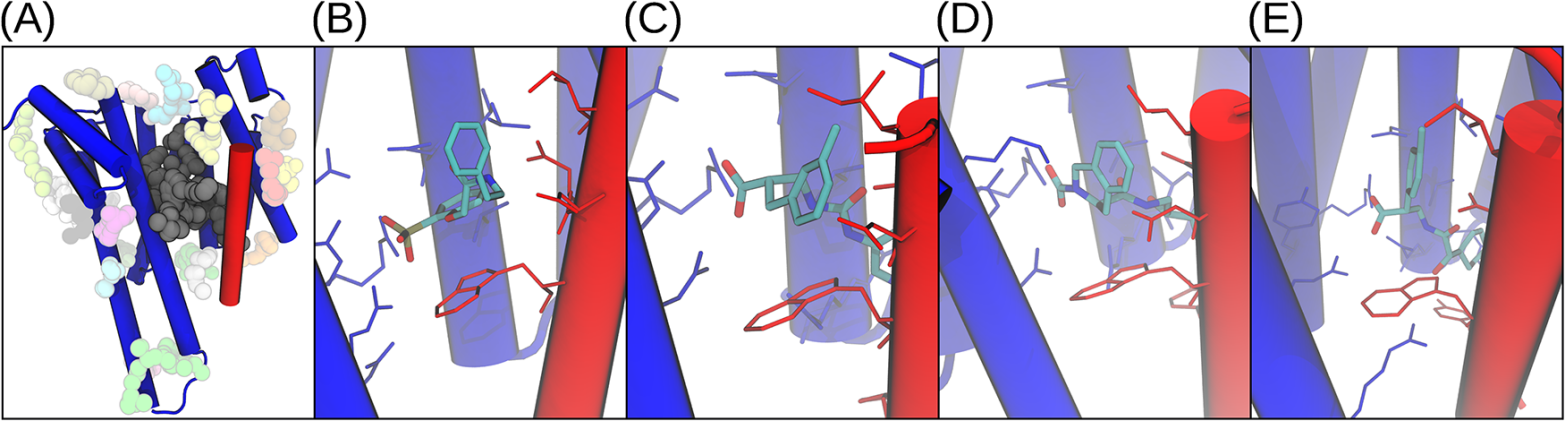
Pharmacophore hits without prior pocket information (4gnt). (A) Pocket identification using Fpocket.^29^ Fpocket predicts multiple potential pockets, shown as faded colored spheres. Treating the pocket probe as a ligand, the pocket with the highest harmonic mean of buried surface area with respect to the two protein chains is selected as the binding pocket and highlighted by gray spheres. (B) The experimentally known ligand emerges as one of the top hits when included in the ligand database and, after 10 ns of simulation, adopts a pose very close to the experimentally determined structure. Protein chain residues within 5 Å of the ligand are shown as sticks in the corresponding colors. (C–E) Poses of the top three ligands with the highest MMGBSA scores (Figure S30B) at the end of the 10 ns simulations. The protein residues in the vicinity of these ligands closely resemble those interacting with the known ligand, suggesting a similar mode of interaction.

## Discussion

PPI stabilization by drug-like compounds provides a promising concept to modulate protein functions broadening the range of druggable targets. While computational methods are effective for systematic ligand screening, they have been less explored for PPI stabilization compared to inhibitor–based approaches. Here, we devised a pharmacophore-screening method, in which pharmacophores are generated from all-atom MD simulations of the complex in the absence of a stabilizer. Pharmacophore features were identified by analyzing the interactions of water molecules and ions with binding-site residues across the MD trajectory, and their positions were assigned from a representative snapshot, so that the resulting hits are already in a docked conformation suitable for MD-based rescoring. Application to seven important systems with known stabilizers shows that our method can identify ligands with MMGBSA or Boltz-2 scores overall comparable to those of the known stabilizers.

Previous methods, including PyRod^27^ and water pharmacophore approaches,^28^ have primarily been evaluated in retrospective benchmarking studies and generally considered only a limited number of pharmacophore models. Moreover, these models function mainly as filters: hits identified must undergo separate docking to generate plausible binding poses for further scoring.^62^ In contrast, our prospective approach systematically ranks and screens hundreds of thousands of pharmacophore models using Pharmer,^20^ allowing efficient identification of effective hits already in docked, pharmacophore-aligned conformations. (Figure 4).

Many ligands stabilize PPIs through a dual-binding mechanism, where the ligand exhibits nearly equal interactions to both protein partners.^9^ Our workflow enforces this condition in the pharmacophore model score as well as in the filtering stage in the form of harmonic means of BSAs with respect to the two protein partners. As a result, PP complexes such as 5ad3 and 3b6q, which are known to be stabilized via the dual-binding mechanism, also display similar behavior with many of the top pharmacophore hits (Figure 3). Overall, the stabilization modes of the top pharmacophore hits closely resemble those of the known ligands (Figure 3).

Filtering pharmacophore hits by ligand BSA appears to be a simple yet effective way to obtain ligands with often high MMGBSA scores. System–specific adjustments can further improve performance; for example, in 5ad3, filtering pharmacophore models during screening based on feature pair distances yielded a higher fraction of top-scoring ligands. These adjustments can be easily implemented in our workflow with simple Python functions. Rescoring with MMGBSA also has inherent limitations due to the underlying approximations such as the neglect of entropic contributions from both the ligand and the protein, as well as the use of an implicit solvent model. Consequently, the correlation with experiments has been shown to exhibit a system dependent behavior.^63,64^ However, ranking different ligands binding to the same pocket, as done in our case, is generally more reliable and often provides meaningful relative comparisons.^63^ More generally, the efficient and accurate estimation of protein–ligand binding free energies remains an open challenge. While more rigorous approaches, such as free energy perturbation or alchemical transformations, can offer higher accuracy, they are typically prohibitively expensive when applied to large ligand sets. Given these limitations, the hits obtained from our workflow can be further filtered using alternative scoring methods, not limited to MMGBSA. Rescoring using deep learning–based methods such as Boltz-2 yielded higher scores for most BSA–filtered ligands compared to known ligands and both Boltz-2 and AF3 positioned the majority of ligands in the same pocket as the pharmacophore model, further underscoring the effectiveness of BSA filtering. However, binder probabilities remained consistently higher for known ligands, likely reflecting the model’s training on known complexes and its tendency to memorize them.^65,66^ Furthermore, all the known ligands satisfy multiple pharmacophore models and appear in the hit list, but the BSA filter does not always rank them highest for MMGBSA rescoring. Thus, BSA filtering is quick and very useful but not perfect. Machine learning based scoring methods can improve the filtering e.g., methods like GEMS^67^ which predict affinity are more suitable. However, such scoring methods can predict only an overall binding affinity of the ligand to the whole complex, developing models that also decompose contributions of individual ligand–protein partner interactions could help identify more potent stabilizers from the existing hits.

Finally, transient pockets or cryptic sites that emerge during MD simulations^9^ can be directly exploited by selecting and analyzing frames of interest from the same trajectories. This enables the identification of stabilizers tailored to specific conformational states. Even when no binding pocket information is available, we can identify potent stabilizers by first predicting the pocket using a pocket detection tool such as Fpocket.^29^ Nevertheless, having prior approximate knowledge of the binding site allows for a more focused and efficient screening, increasing the likelihood of discovering more effective stabilizers. Coupled with recent advances in PP complex structure prediction,^68^ the workflow presented here offers a powerful and generalizable strategy for discovering small molecules capable of modulating protein interactions across a wide range of biological and therapeutic applications.

## Supplementary Material



## Data Availability

All required code, along with a database of 10 million randomly selected compounds, is publicly available at https://github.com/ibrahim-mohd/PPIS-MDPharma, enabling rapid application of the workflow for different use cases.

## Notes

Plots of pharmacophore features for each complex; and chemical structures of the top ligands and their ZINC IDs and rescoring results from Boltz-2 (PDF)
