# Supplementary material for: Protein–Protein Interaction Stabilizers from MD Simulation-Derived Pharmacophores
Source: J Chem Inf Model. 2026 May 1;66(10):6131–40. doi: 10.1021/acs.jcim.6c00290 (PMC13213832; doi:10.1021/acs.jcim.6c00290)
Supplement: Supplementary file 1 [file ci6c00290_si_001.pdf]

Supplementary:Protein-Protein Interaction Stabilizers from MD Simulation-derived Pharmacophores

Mohd Ibrahim and Martin Zacharias*

Physics Department and Center for Functional Protein Assemblies, Technical University of Munich, 85748 Garching, Germany

E-mail: zacharias@tum.de

Contents

1 Pharmacophore features for different systems from MD simulations S2

2 Chemical structures and ZINC IDs of top stabilizers S8

3 Rescoring and filtering with Boltz-2 S15

3.1 Rescoring MMGBSA scored ligands with Boltz-2 . . . . S15

3.2 Boltz-2 vs AlphaFold-3 (AF3) placement of pharmacophore hits S20

3.3 Filtering ligands with Boltz-2 . . . . S22

4 Finding ligands without prior pocket information: detect binding pocket using Fpocket S27

References S29

1 Pharmacophore features for different systems from MD simulations

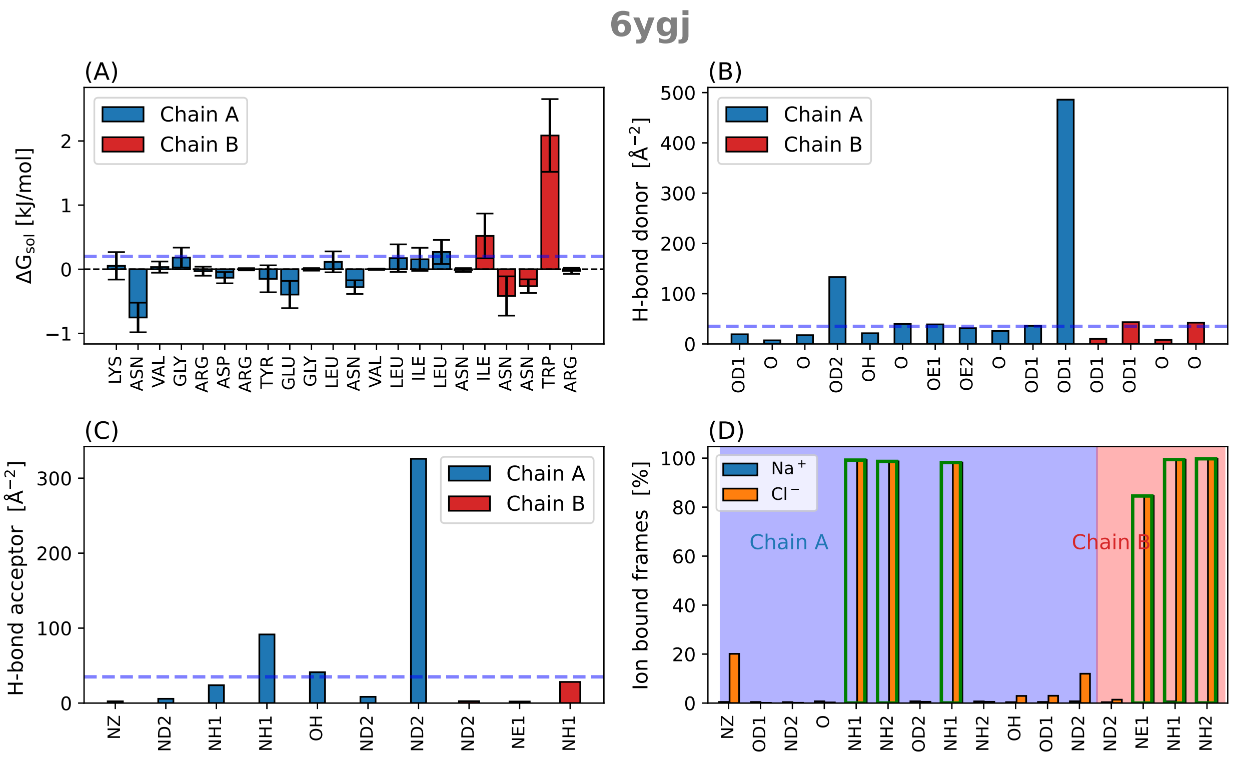

Figure S1: **Pharmacophore features from MD simulation (6ygj case).** (A) Solvation free energy estimation for each pocket residue to identify hydrophobic and aromatic regions. (B) H-bond donor (C) H-bond acceptor frequencies normalized to SASA. All sites in A-C with values above the dashed blue horizontal threshold line are considered a pharmacophore feature. (D) Cation (Na^+) and anion (Cl^-) accumulation sites. The bars indicate the percentage of MD frames in which an anion or cation comes in contact with a given atoms in the pocket. A contact is defined when distance between ion and the pocket atoms is < 4 Å. The green rectangular outlines indicate sites which are above the pharmacophore feature threshold and are considered as a feature. In this case, there are 14 features in the master pharmacophore model. Aromatic = 1 (chainA=0, chainB=1), Hydrophobic= 2 (chainA=1, chainB=1), Hydrogen bond donor=7 (chainA=5, chainB=2), Hydrogen bond acceptor=3 (chainA=3, chainB=0) and Negative ion site =1 (chainA=1, chainB=0). Note that even though we have 6 negative ion sites in (D) but only have one negative ion site in the master pharmacophore model. This is because, all the six sites are coordinated to the same Cl^- ion leading to only one negative ion site with a very high score. It is a direct consequence of obtaining the pharmacophore coordinates from bound ions and is not hard coded.

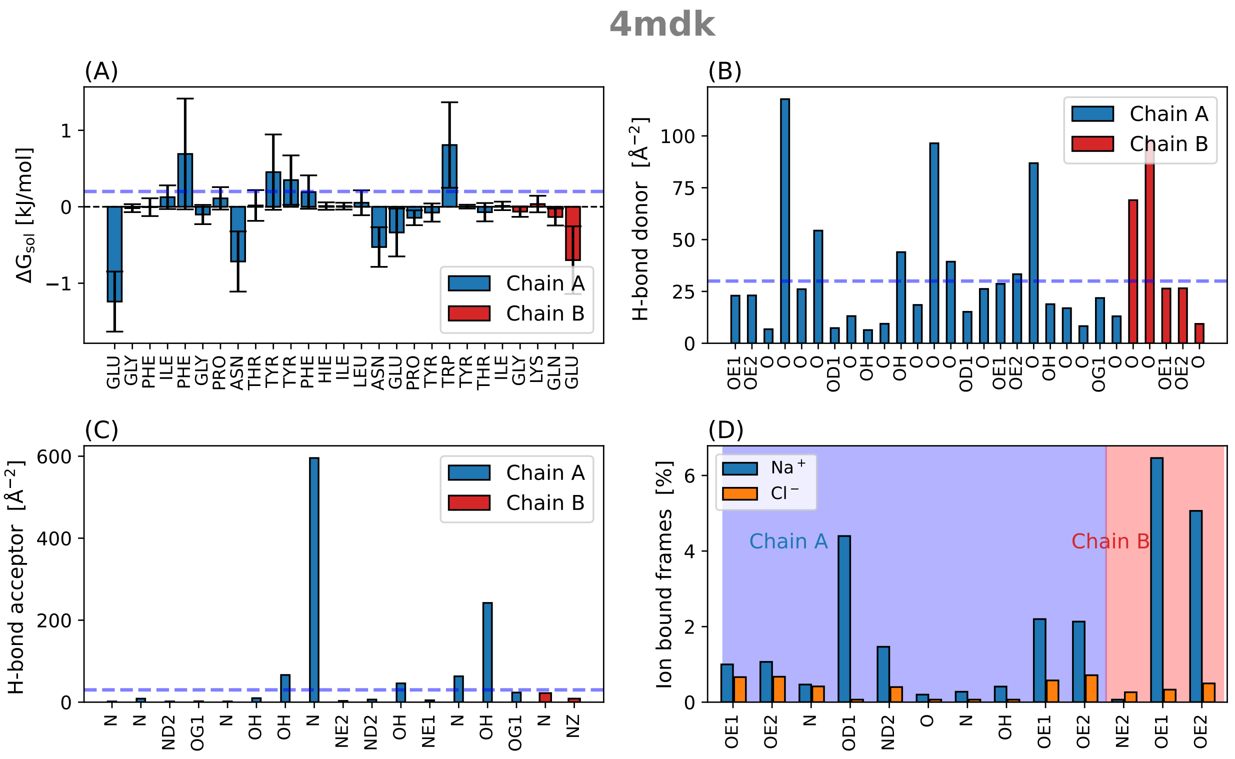

Figure S2: **Pharmacophore features from MD simulation (4mdk case).** (A-D) Same as in Figure S1. There are 18 features in the master pharmacophore model. Aromatic = 4 (chainA=4, chainB=0), Hydrogen bond donor=9 (chainA=7, chainB=2), Hydrogen bond acceptor 5 (chainA=5, chainB=0).

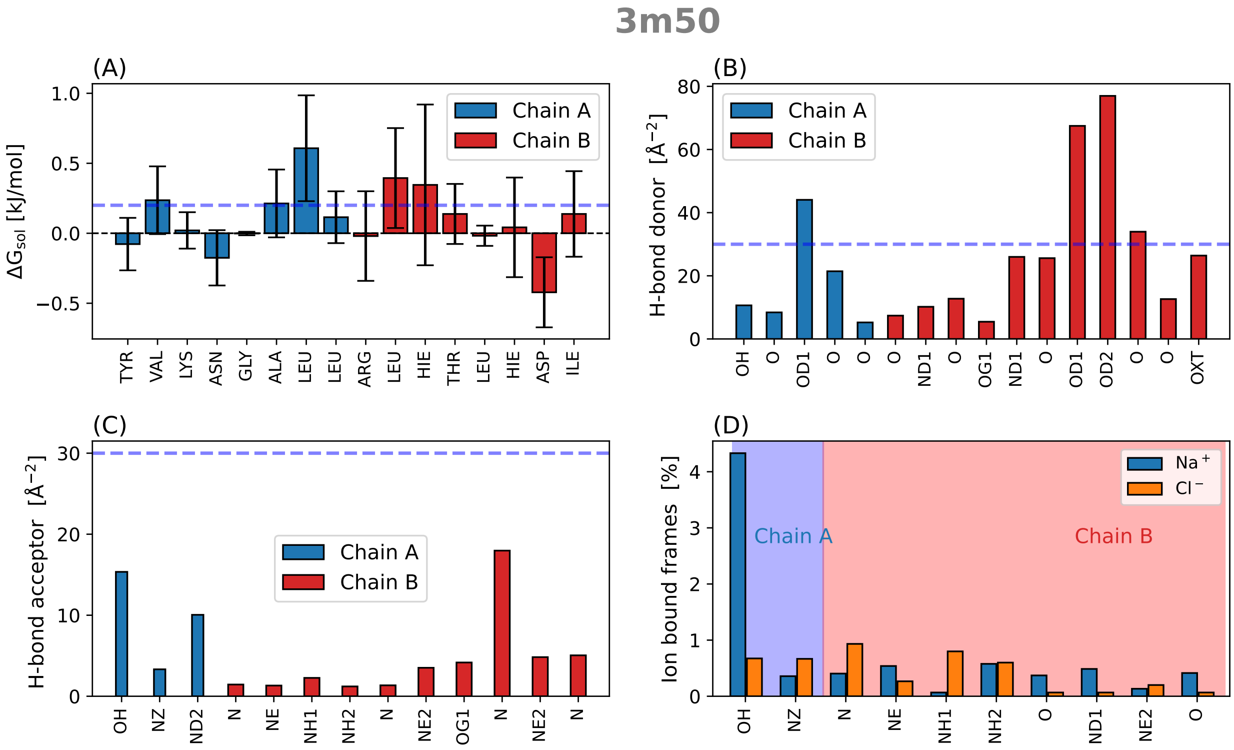

Figure S3: **Pharmacophore features from MD simulation (3m50 case).** (A-D) Same as in Figure S1. There are 9 features in the master pharmacophore model. Hydrophobic = 5 (chainA=3, chainB=2), Hydrogen bond donor=4 (chainA=1, chainB=3).

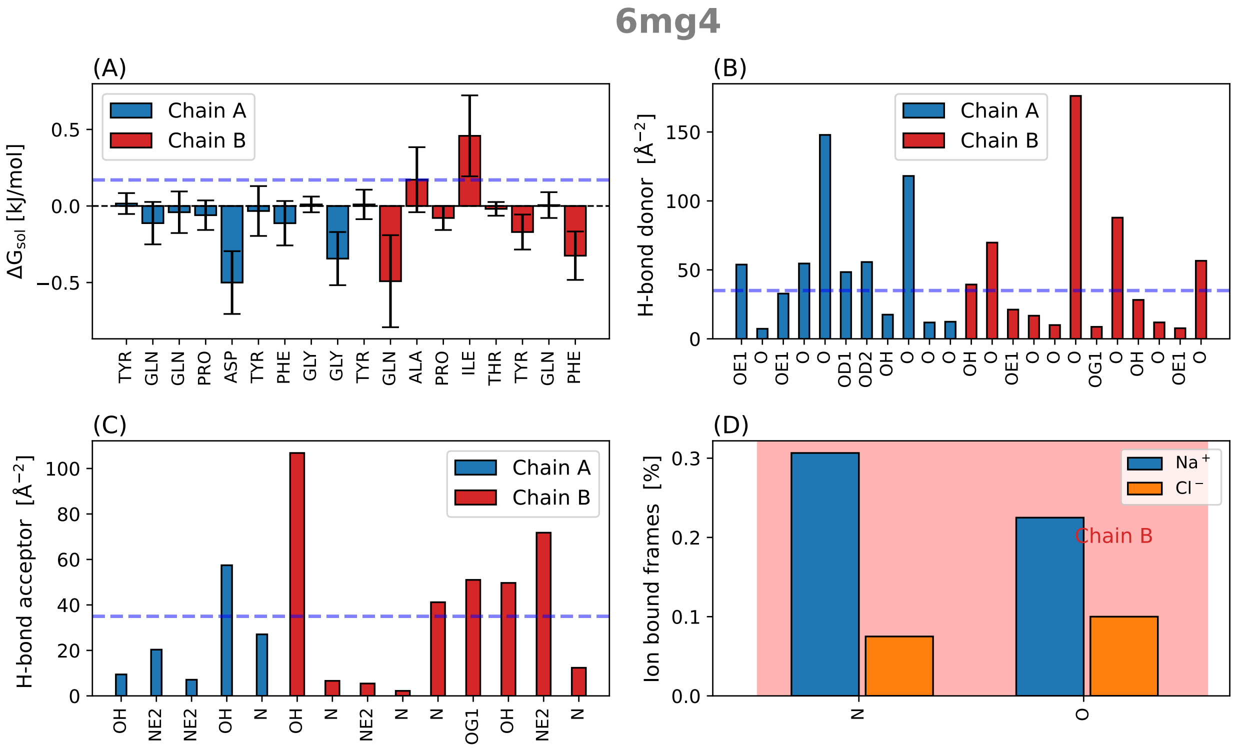

Figure S4: **Pharmacophore features from MD simulation (6mg4).** (A-D) Same as in Figure S1. There are 18 features in the master pharmacophore model. Hydrophobic =2 (chainA=0, chainB=2), Hydrogen bond donor=10 (chainA=6, chainB=4), Hydrogen bond acceptor= 6 (chainA=1, chainB=5).

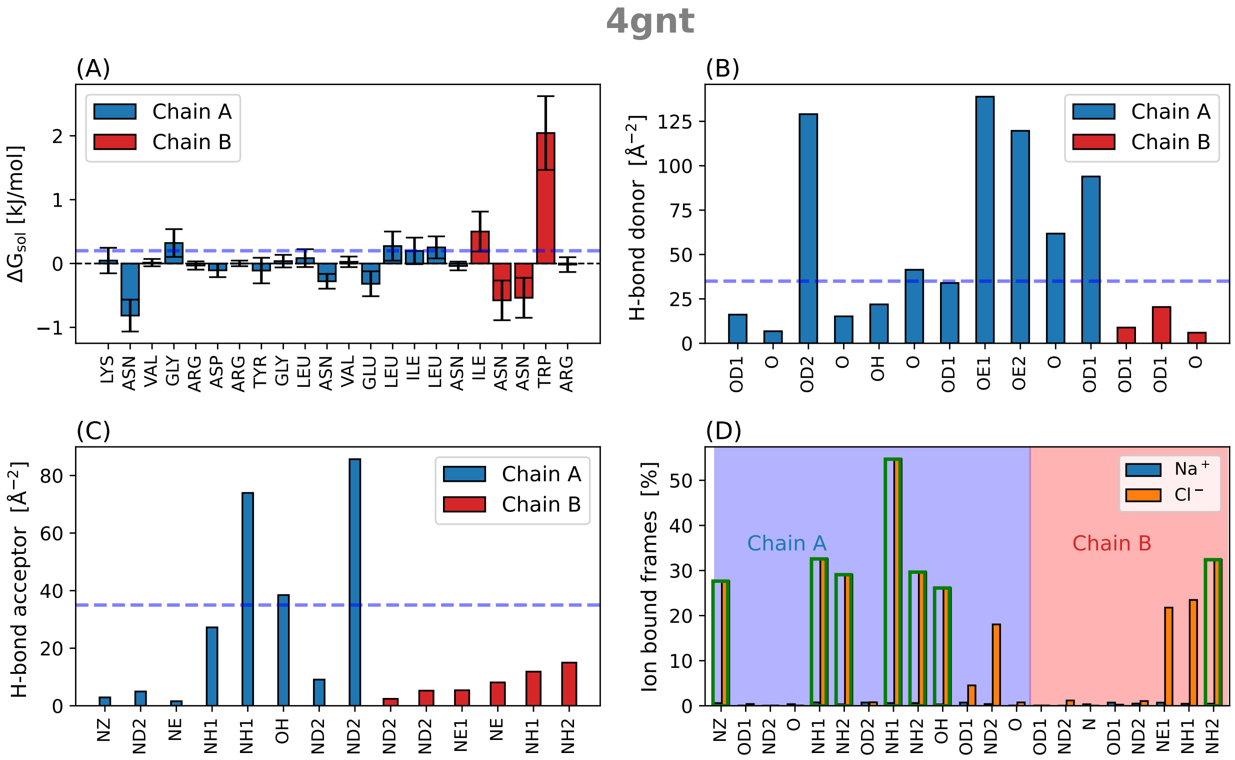

Figure S5: **Pharmacophore features from MD simulation (4gnt).** (A-D) Same as in Figure S1. There are: There are 16 features in the master pharmacophore model. Aromatic = 1 (chainA=0, chainB=1), Hydrophobic= 4 (chainA=3, chainB=1), Hydrogen bond donor=6 (chainA=6, chainB=0), Hydrogen bond acceptor=3 (chainA=3, chainB=0) and Negative ion site =2 (chainA=2, chainB=0).

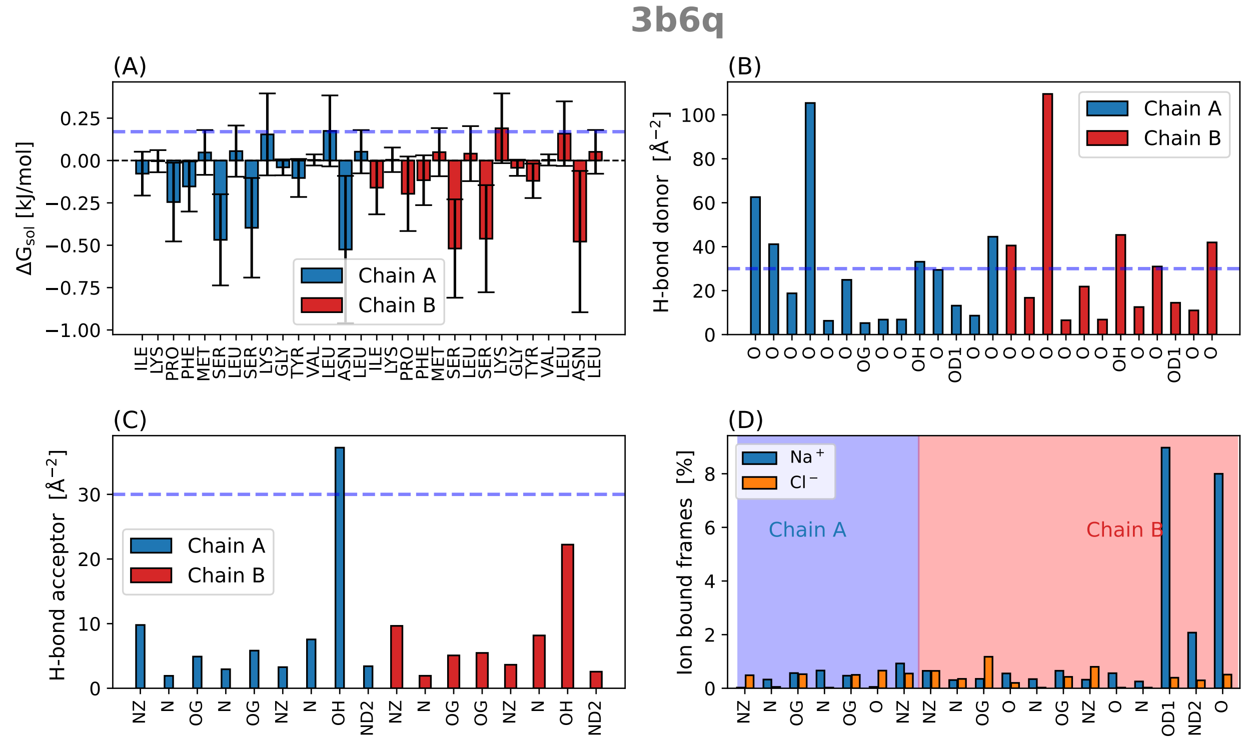

Figure S6: **Pharmacophore features from MD simulation (3b6q).** (A-D) Same as in Figure S1. There are: There are 11 features in the master pharmacophore model. Hydrophobic= 2 (chainA=1, chainB=1), Hydrogen bond donor=8 (chainA=5, chainB=3), Hydrogen bond acceptor=1 (chainA=1, chainB=0). Note that there are 10 hydrogen bond donor sites above the threshold line. However, two of them are ignored since for these cases no water molecule was found in hydrogen bonding geometry in the chosen frame. One can also consider them with the corresponding flag in our workflow.

2 Chemical structures and ZINC IDs of top stabilizers

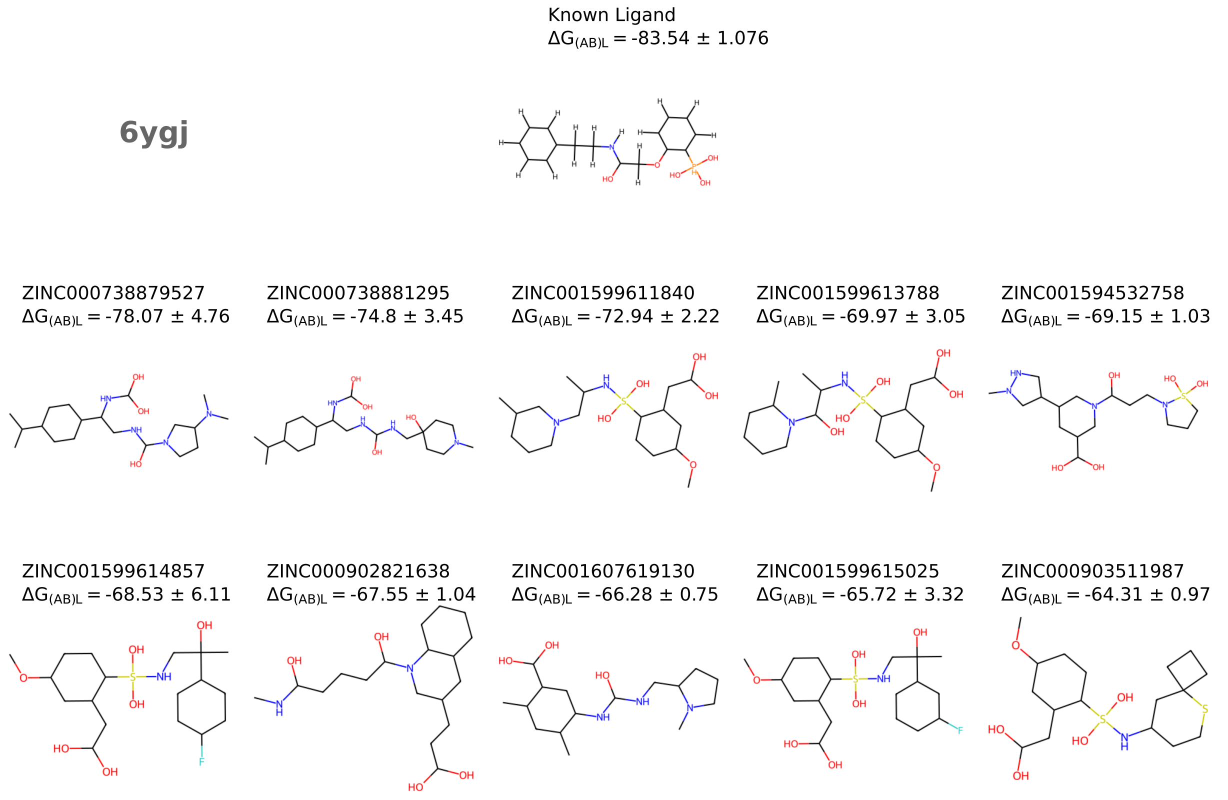

Figure S7: **Top ligands for 6ygj:** ZINC ID and chemical structures of top ligands shown in Figure 3 of the main text. The known experimental ligand is also displayed at the top. For each ligand the MMGBSA binding free energy ($\Delta G_{(AB)L}$) of the ligand with the whole protein-protein complex is shown.

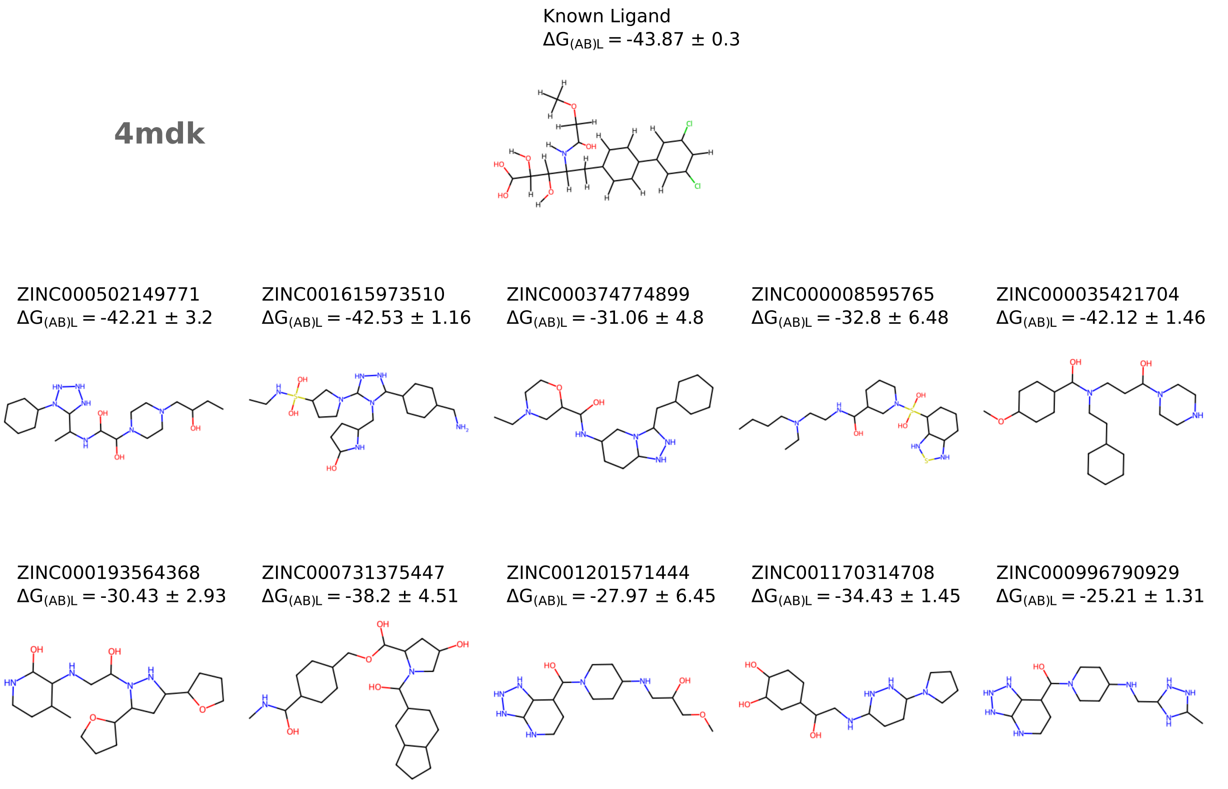

Figure S8: **Top ligands for 4mdk:** ZINC ID and chemical structures of top ligands shown in Figure 3 of the main text. The known experimental ligand is also displayed at the top. For each ligand the MMGBSA binding free energy ($\Delta G_{(AB)L}$) of the ligand with the whole protein-protein complex is shown.

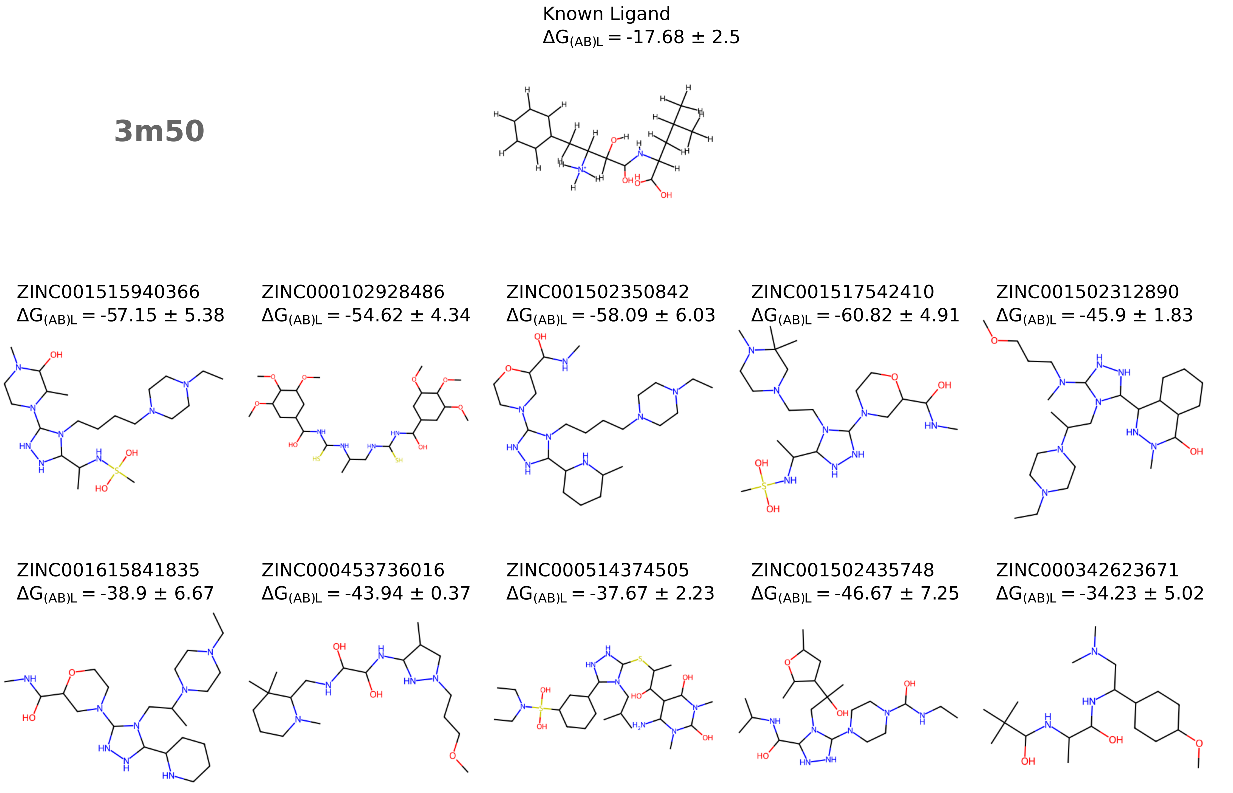

Figure S9: **Top ligands for 3m50:** ZINC ID and chemical structures of top ligands shown in Figure 3 of the main text. The known experimental ligand is also displayed at the top. For each ligand the MMGBSA binding free energy ($\Delta G_{(AB)L}$) of the ligand with the whole protein-protein complex is shown.

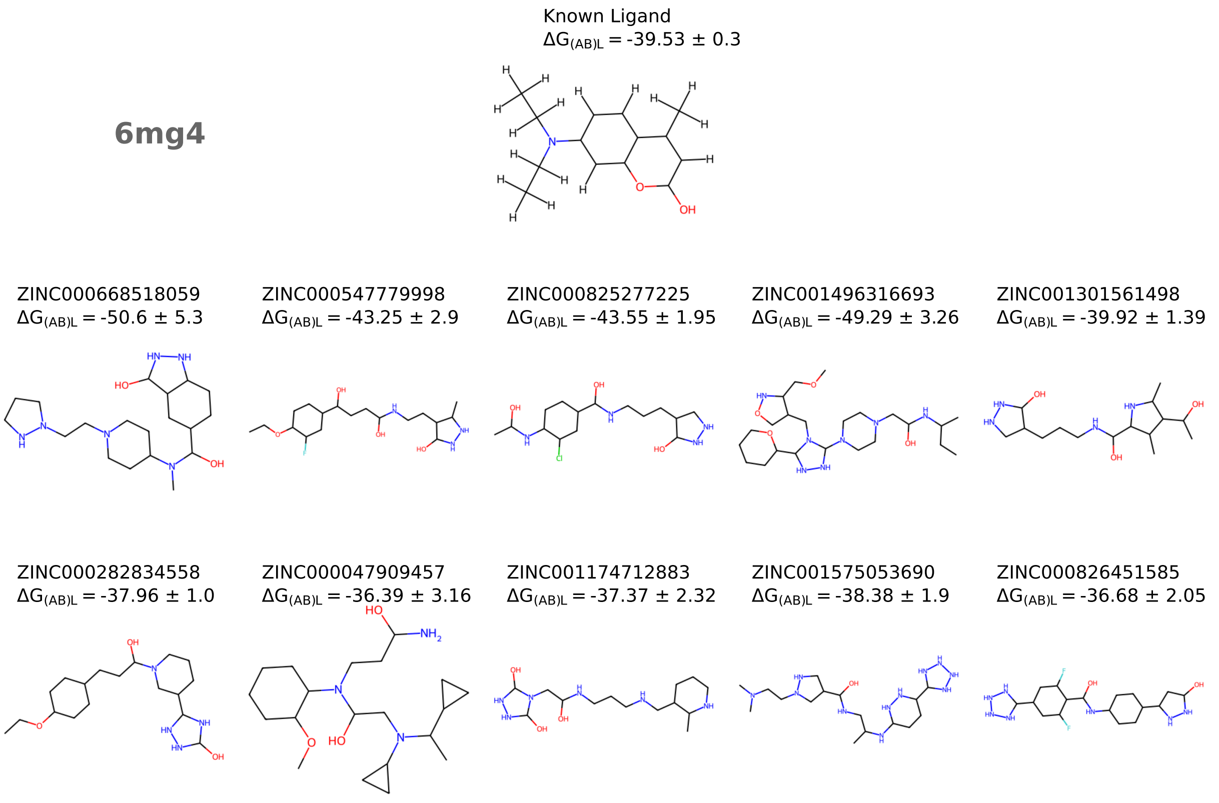

Figure S10: **Top ligands for 6mg4:** ZINC ID and chemical structures of top ligands shown in Figure 3 of the main text. The known experimental ligand is also displayed at the top. For each ligand the MMGBSA binding free energy ($\Delta G_{(AB)L}$) of the ligand with the whole protein-protein complex is shown.

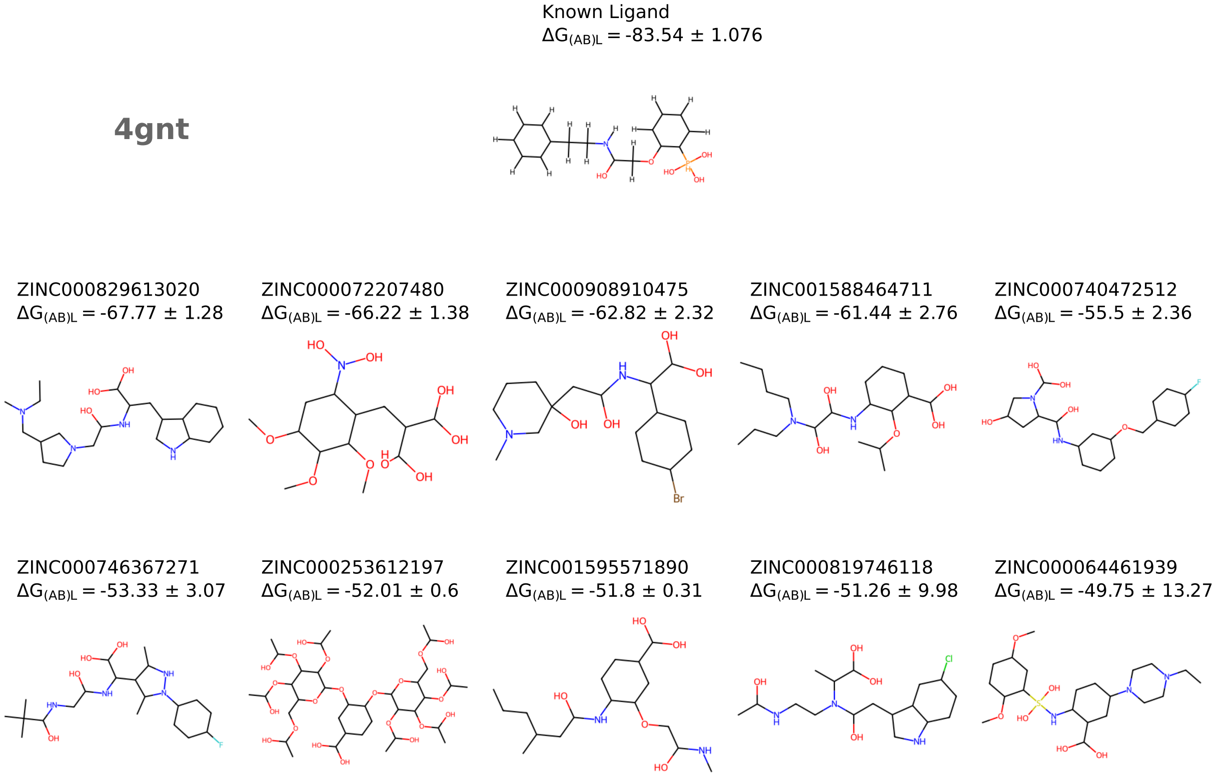

Figure S11: **Top ligands for 4gnt:** ZINC ID and chemical structures of top ligands shown in Figure 3 of the main text. The known experimental ligand is also displayed at the top. For each ligand the MMGBSA binding free energy ($\Delta G_{(AB)L}$) of the ligand with the whole protein-protein complex is shown.

3b6q

Known Ligand
ΔG(AB)L = -47.85 ± 2.1

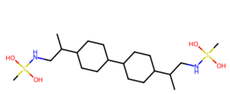

ZINC001502429240
ΔG(AB)L = -47.55 ± 0.9

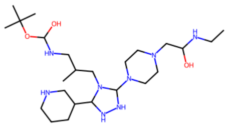

ZINC000026468553
ΔG(AB)L = -49.42 ± 4.96

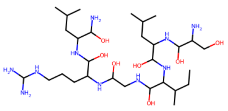

ZINC000101318345
ΔG(AB)L = -45.72 ± 5.61

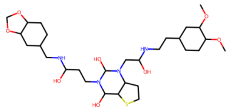

ZINC001502316500
ΔG(AB)L = -46.43 ± 7.8

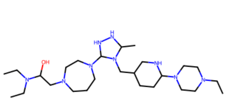

ZINC001503099700
ΔG(AB)L = -45.19 ± 5.98

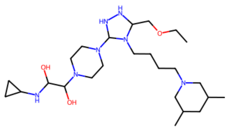

ZINC000150344983
ΔG(AB)L = -40.79 ± 1.88

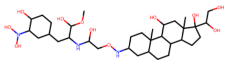

ZINC001509223996
ΔG(AB)L = -40.8 ± 2.0

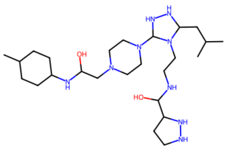

ZINC001508381953
ΔG(AB)L = -40.88 ± 1.93

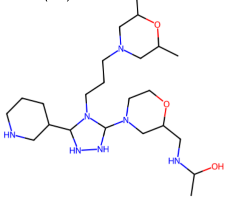

ZINC001502258207
ΔG(AB)L = -39.85 ± 8.75

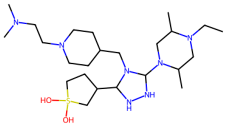

ZINC000952869025
ΔG(AB)L = -46.79 ± 4.66

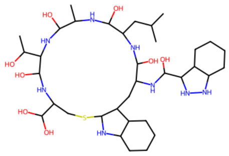

Figure S12: **Top ligands for 3b6q:** ZINC ID and chemical structures of top ligands shown in Figure 3 of the main text. The known experimental ligand is also displayed at the top. For each ligand the MMGBSA binding free energy ($\Delta G_{(AB)L}$) of the ligand with the whole protein-protein complex is shown.

Known Ligand
ΔG$_{(AB)L}$ = -50.87 ± 1.5

5ad3

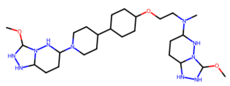

ZINC000150456687
ΔG$_{(AB)L}$ = -63.5 ± 2.89

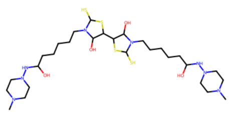

ZINC000150406958
ΔG$_{(AB)L}$ = -65.85 ± 1.69

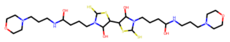

ZINC000095787653
ΔG$_{(AB)L}$ = -61.53 ± 5.61

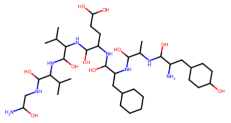

ZINC000263583904
ΔG$_{(AB)L}$ = -56.66 ± 5.06

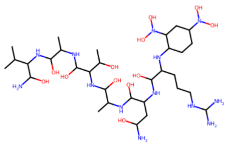

ZINC000252441406
ΔG$_{(AB)L}$ = -55.75 ± 2.22

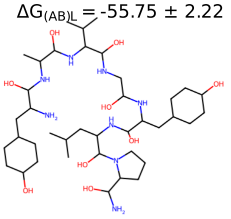

ZINC000095543647
ΔG$_{(AB)L}$ = -52.54 ± 5.52

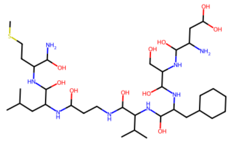

ZINC000217346611
ΔG$_{(AB)L}$ = -52.58 ± 2.55

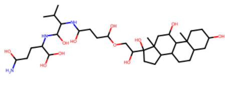

ZINC000095628075
ΔG$_{(AB)L}$ = -57.29 ± 1.33

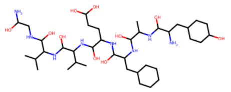

ZINC000082178671
ΔG$_{(AB)L}$ = -48.79 ± 1.98

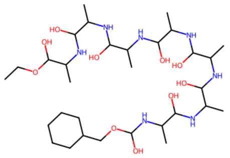

ZINC000644163958
ΔG$_{(AB)L}$ = -49.44 ± 9.0

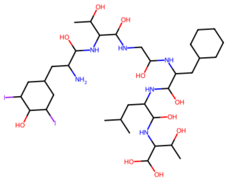

Figure S13: **Top ligands for 5da3:** ZINC ID and chemical structures of top ligands shown in Figure 3 of the main text. The known experimental ligand is also displayed at the top. For each ligand the MMGBSA binding free energy (ΔG$_{(AB)L}$) of the ligand with the whole protein-protein complex is shown.

3 Rescoring and filtering with Boltz-2

The ligands scored with MMGBSA were re-scored using Boltz-2[1] by using the PP complex sequences and ligand smiles string as input. For each PP-ligand complex we obtain the (i) Boltz-2 predicted affinity of the ligand to the whole complex (ii) the binder probability of the ligand (iii) the RMSD of predicted PP complex with the energy minimized experimental structure (iv) the overlap between the pocket residues of Boltz-2 and that from pharmacophore screening. The overlap between the ligand pocket predicted by Boltz-2 and pharmacophore model is calculated by

$$\text{Overlap} = \frac{len(\text{Boltz-2 Pocket Residue IDs} \cap \text{Pharma Pocket Residue IDs})}{\min(len(\text{Boltz-2 Pocket Residue IDs}), len(\text{Pharma Pocket Residue IDs}))} \quad (1)$$

The overlap is 0 when there is no common residue and 1 when one pocket is a subset of other.

3.1 Rescoring MMGBSA scored ligands with Boltz-2

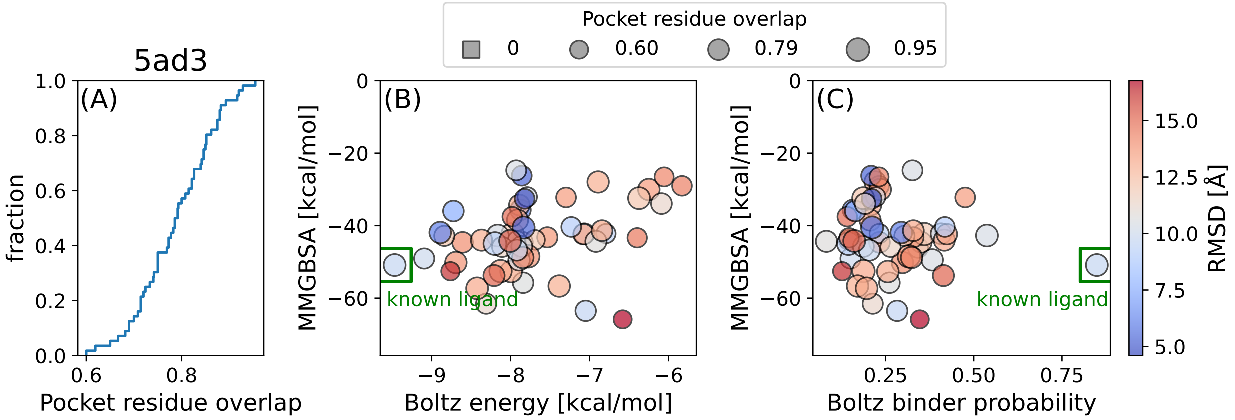

Figure S14: **Rescoring with Boltz-2 (5ad3):** (A) Overlap between pocket residues predicted by Boltz-2 and pharmacophore hits. (B) MMGBSA binding free energy ($\Delta G_{(AB)L}$) plotted against the Boltz-2 affinity score. Marker size reflects the pocket overlap, with larger circles indicating greater overlap. Square markers denote cases with zero pocket overlap. Marker color indicates the RMSD between the Boltz-2-predicted protein–protein complex and the energy-minimized experimental structure. (C) MMGBSA binding free energy versus the Boltz-2-predicted binder probability (ranging from 0 to 1). The marker corresponding to the experimentally known ligand is highlighted with a green square.

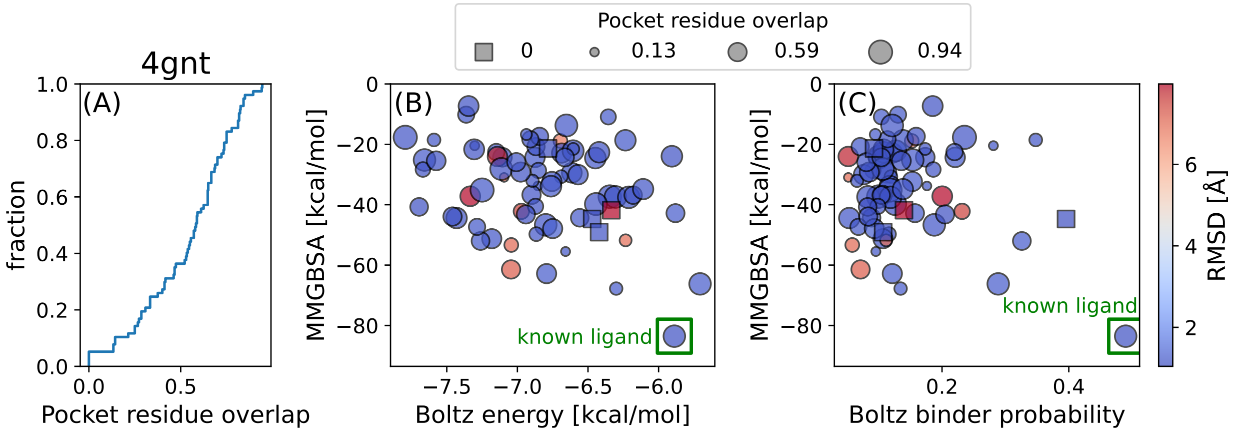

Figure S15: **Rescoring with Boltz-2 (4gnt):** (A) Overlap between pocket residues predicted by Boltz-2 and pharmacophore hits. (B) MMGBSA binding free energy ($\Delta G_{(AB)L}$) plotted against the Boltz-2 affinity score. Marker size reflects the pocket overlap, with larger circles indicating greater overlap. Square markers denote cases with zero pocket overlap. Marker color indicates the RMSD between the Boltz-2-predicted protein–protein complex and the energy-minimized experimental structure. (C) MMGBSA binding free energy versus the Boltz-2-predicted binder probability (ranging from 0 to 1). The marker corresponding to the experimentally known ligand is highlighted with a green square.

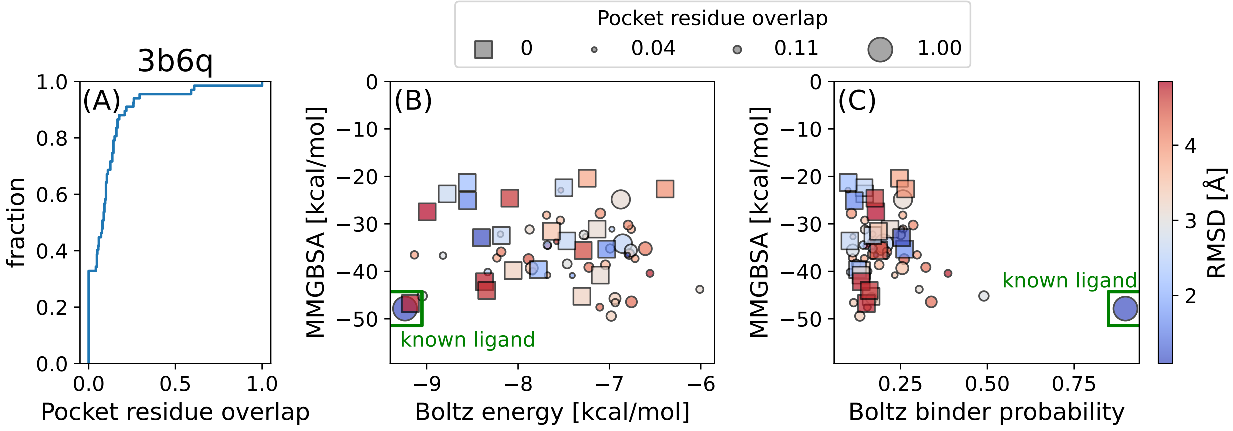

Figure S16: **Rescoring with Boltz-2 (3b6q):** (A) Overlap between pocket residues predicted by Boltz-2 and pharmacophore hits. (B) MMGBSA binding free energy ($\Delta G_{(AB)L}$) plotted against the Boltz-2 affinity score. Marker size reflects the pocket overlap, with larger circles indicating greater overlap. Square markers denote cases with zero pocket overlap. Marker color indicates the RMSD between the Boltz-2-predicted protein–protein complex and the energy-minimized experimental structure. (C) MMGBSA binding free energy versus the Boltz-2-predicted binder probability (ranging from 0 to 1). The marker corresponding to the experimentally known ligand is highlighted with a green square.

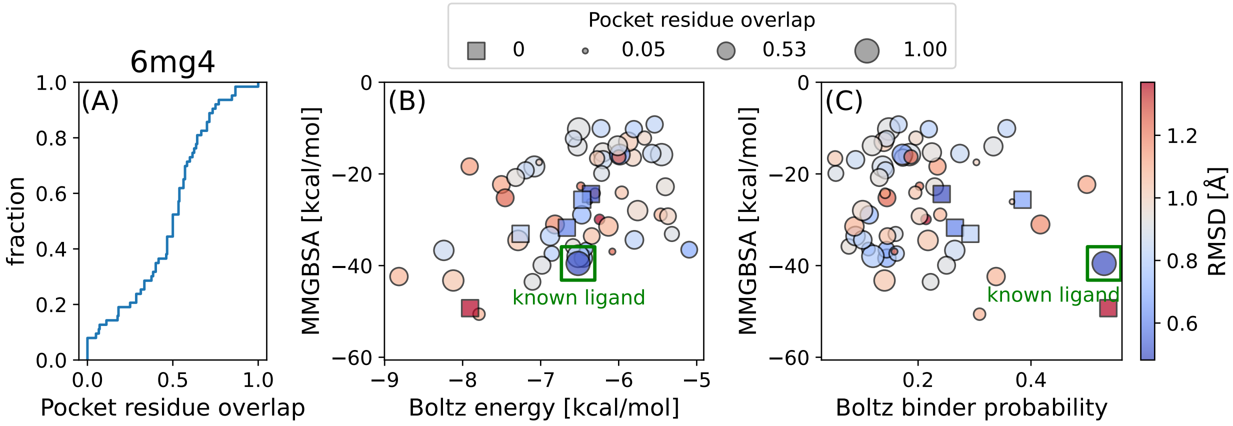

Figure S17: **Rescoring with Boltz-2 (6mg4):** (A) Overlap between pocket residues predicted by Boltz-2 and pharmacophore hits. (B) MMGBSA binding free energy ($\Delta G_{(AB)L}$) plotted against the Boltz-2 affinity score. Marker size reflects the pocket overlap, with larger circles indicating greater overlap. Square markers denote cases with zero pocket overlap. Marker color indicates the RMSD between the Boltz-2-predicted protein–protein complex and the energy-minimized experimental structure. (C) MMGBSA binding free energy versus the Boltz-2-predicted binder probability (ranging from 0 to 1). The marker corresponding to the experimentally known ligand is highlighted with a green square.

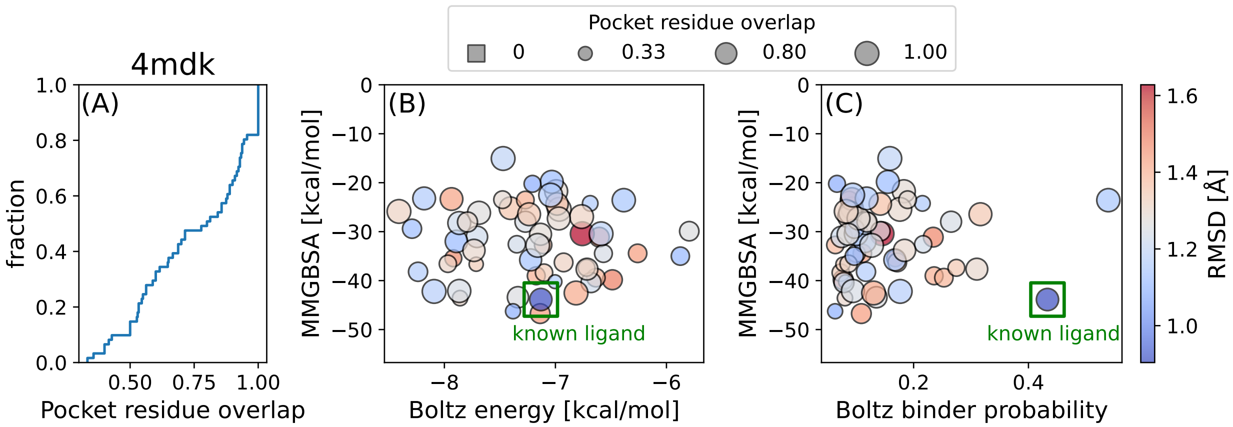

Figure S18: **Rescoring with Boltz-2 (4mdk):** (A) Overlap between pocket residues predicted by Boltz-2 and pharmacophore hits. (B) MMGBSA binding free energy ($\Delta G_{(AB)L}$) plotted against the Boltz-2 affinity score. Marker size reflects the pocket overlap, with larger circles indicating greater overlap. Square markers denote cases with zero pocket overlap. Marker color indicates the RMSD between the Boltz-2-predicted protein–protein complex and the energy-minimized experimental structure. (C) MMGBSA binding free energy versus the Boltz-2-predicted binder probability (ranging from 0 to 1). The marker corresponding to the experimentally known ligand is highlighted with a green square.

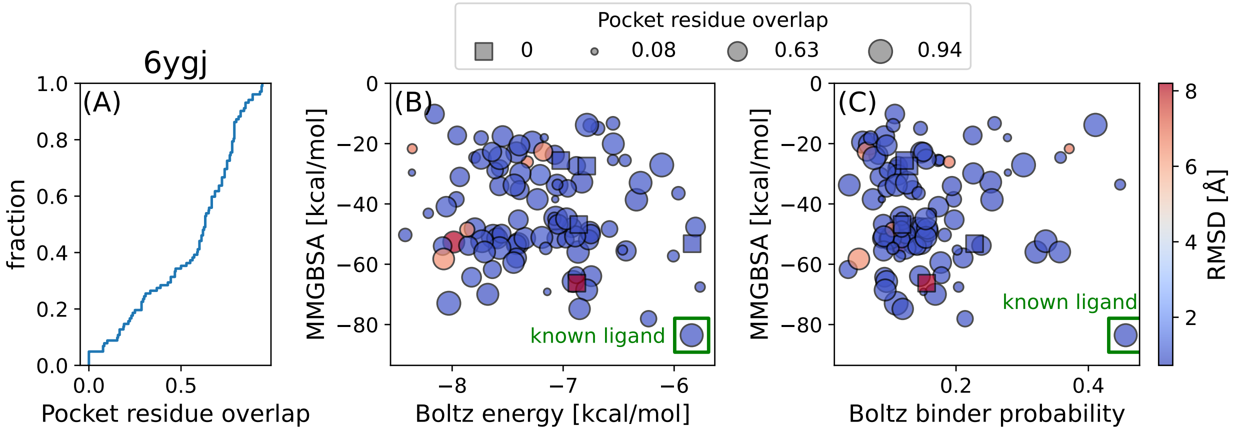

Figure S19: **Rescoring with Boltz-2 (6ygj):** (A) Overlap between pocket residues predicted by Boltz-2 and pharmacophore hits. (B) MMGBSA binding free energy ($\Delta G_{(AB)L}$) plotted against the Boltz-2 affinity score. Marker size reflects the pocket overlap, with larger circles indicating greater overlap. Square markers denote cases with zero pocket overlap. Marker color indicates the RMSD between the Boltz-2-predicted protein–protein complex and the energy-minimized experimental structure. (C) MMGBSA binding free energy versus the Boltz-2-predicted binder probability (ranging from 0 to 1). The marker corresponding to the experimentally known ligand is highlighted with a green square.

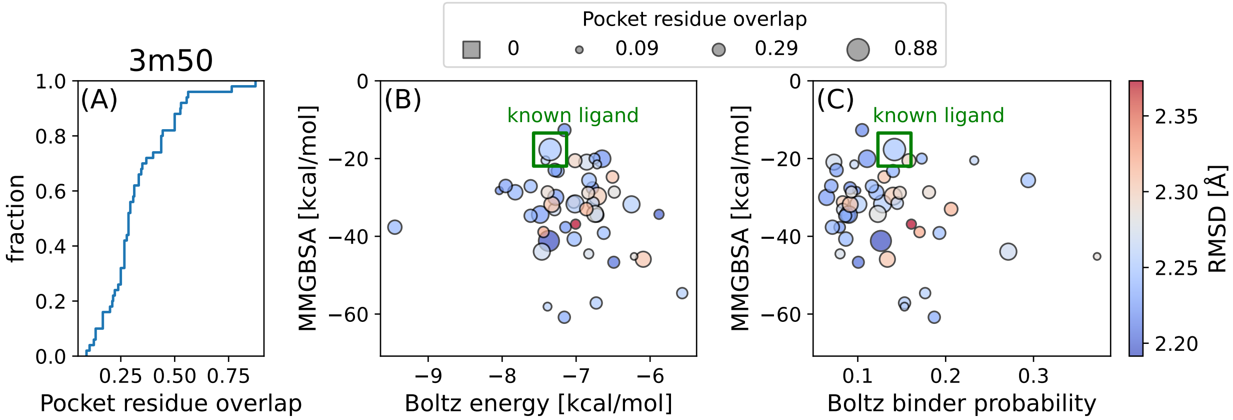

Figure S20: **Rescoring with Boltz-2 (3m50):** (A) Overlap between pocket residues predicted by Boltz-2 and pharmacophore hits. (B) MMGBSA binding free energy ($\Delta G_{(AB)L}$) plotted against the Boltz-2 affinity score. Marker size reflects the pocket overlap, with larger circles indicating greater overlap. Square markers denote cases with zero pocket overlap. Marker color indicates the RMSD between the Boltz-2-predicted protein–protein complex and the energy-minimized experimental structure. (C) MMGBSA binding free energy versus the Boltz-2-predicted binder probability (ranging from 0 to 1). The marker corresponding to the experimentally known ligand is highlighted with a green square.

3.2 Boltz-2 vs AlphaFold-3 (AF3) placement of pharmacophore hits

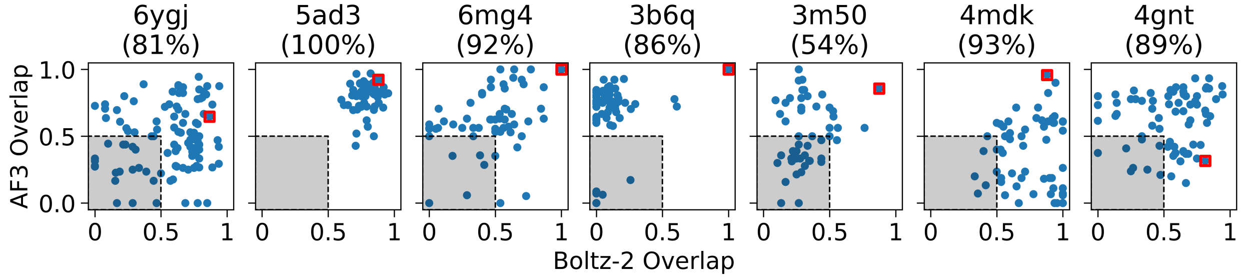

Figure S21: **Boltz-2 Vs AF3 binding site residue overlap correlation:**: Correlation between overlap of ligand binding site residues from AF3 and Boltz-2 with the pharmacophore hits. The highlighted red squares correspond to predictions in presence of the experimentally known stabilizers. The shaded grey region corresponds to ligands that has less than 0.5 overlap from both AF3 and Boltz-2. Outside this region are the ligands exhibiting >0.5 overlap of binding pockets with pharmacophore hits from either of the techniques. The percentage of ligands outside the grey shaded are annotated in the title.

RMSDs of AF3 and Boltz-2 structures in the presence of pharmacophore-hit ligands are generally comparable to holo structures (Figure S22). However, for 5ad3, Boltz-2 deviates by ∼10 Å even with the experimental ligand, with RMSDs reaching ∼16 Å in some cases for both Boltz-2 and AF3 (Figure S22; Figure S23 C, E). These deviations arise from altered relative positioning of protein partners, while individual chains remain structurally consistent with experiment (Figure S23 A-D). A similar trend is observed for 6ygj and its apo form 4gnt (Figure S22, S23 G-H).

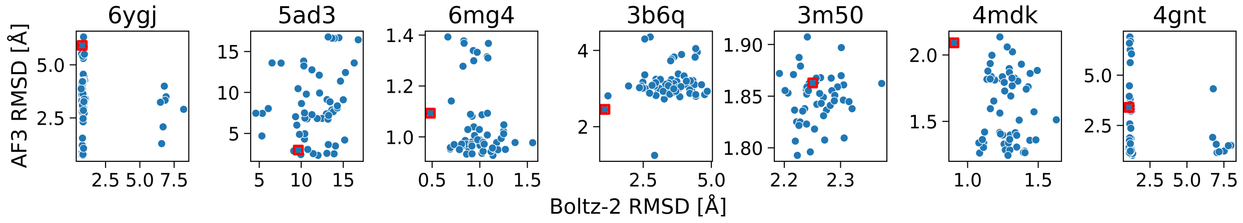

Figure S22: **Boltz-2 vs AF3 RMSD correlation:** The RMSD is calculated between the experimentally known ligand bound form (holo) of the complex and AF3 or Boltz-2 predicted complexes in presence of different pharmacophore hit ligands. The highlighted red squares correspond to predictions in presence of the experimentally known stabilizers.

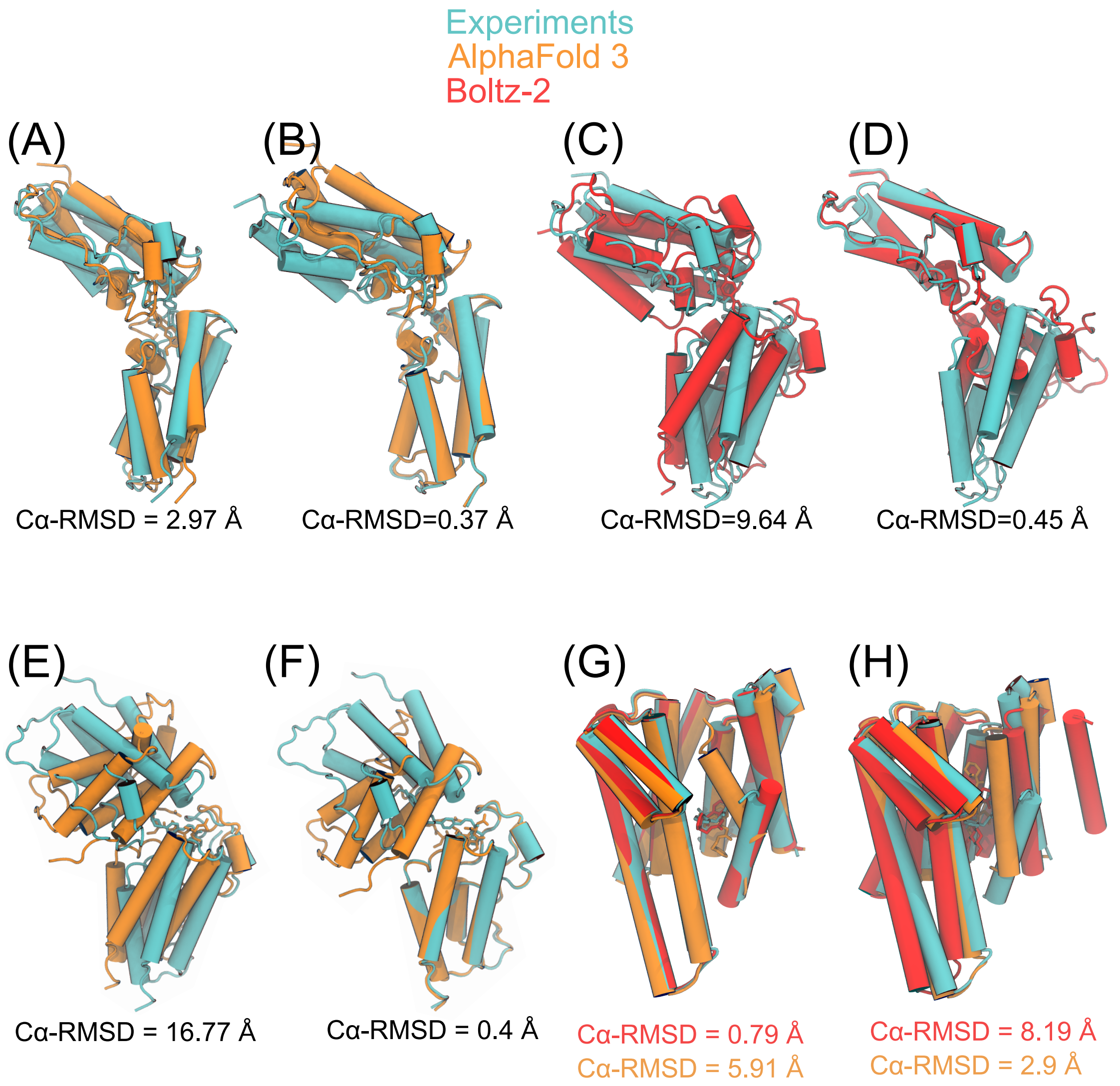

Figure S23: **Representative snapshots of AF3 and Boltz-2 predictions for 5ad3 and 6ygj:** (A–D) Predictions for 5ad3 in the presence of the experimentally known ligand using AF3 and Boltz-2. (A) AF3-predicted structure aligned with the experimental structure; the RMSD is indicated below. (B) Same as (A), but alignment is performed using only a single protein chain (lower chain), with the corresponding RMSD shown. (C) Boltz-2-predicted structure aligned with the experimental structure. (D) Same as (C) but aligned using only one chain of the complex (upper chain). (E–F) AF3 prediction for 5ad3 in the presence of a ligand from the pharmacophore hit. (E) Alignment of the full complex with the experimental structure yields an RMSD of 16.77 Å. (F) Alignment using only the lower chain shows that individual chains closely match the experimental structure. (G–H) Predictions for 6ygj. (G) AF3 and Boltz-2 structures aligned with the experimental structure in the presence of the experimentally known ligand. AF3 deviates due to incorrect positioning of the second chain (ChREBP helix), resulting in a high RMSD. (H) Predictions in the presence of a pharmacophore-hit ligand; Boltz-2 displaces the ChREBP helix (rightmost red helix), leading to an increased RMSD.

3.3 Filtering ligands with Boltz-2

We filtered the pharmacophore hits using two metrics (i) Boltz-2 predicted binding affinity (ii) Boltz-2 predicted binder probability. The top 10-20 highest scoring ligands were re-scored using MMGBSA. The MMGBSA scores for binding affinity filtered and binder probability filtered ligands for 5ad3 are shown in Figure S24 and Figure S25 respectively. For the 4gnt systems only binder affinity filtered ligands were re-scored with MMGBSA (Figure S15).

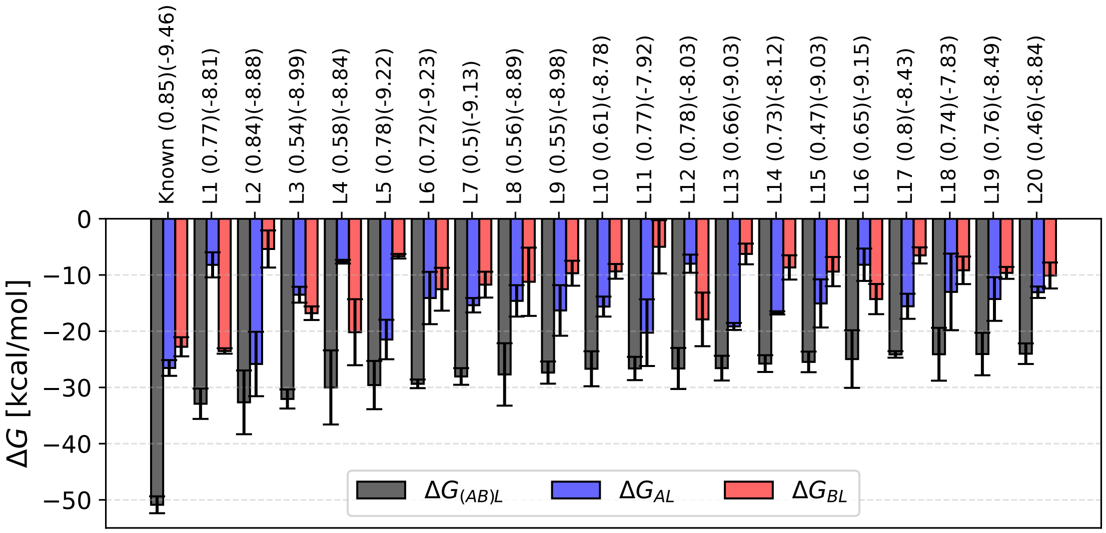

Figure S24: **MMGBSA scores for Boltz-2 binder affinity filtered ligands (5ad3):** MMGBSA free energies of ligand with the protein complex (gray) and with each protein partners (light blue and light red). These ligands were selected for simulation based on their Boltz-2 affinity scores (< -7.7 kcal/mol).. The ticks in the top depicts the Boltz-2 binder probability (lower) and Boltz-2 affinity score (upper).

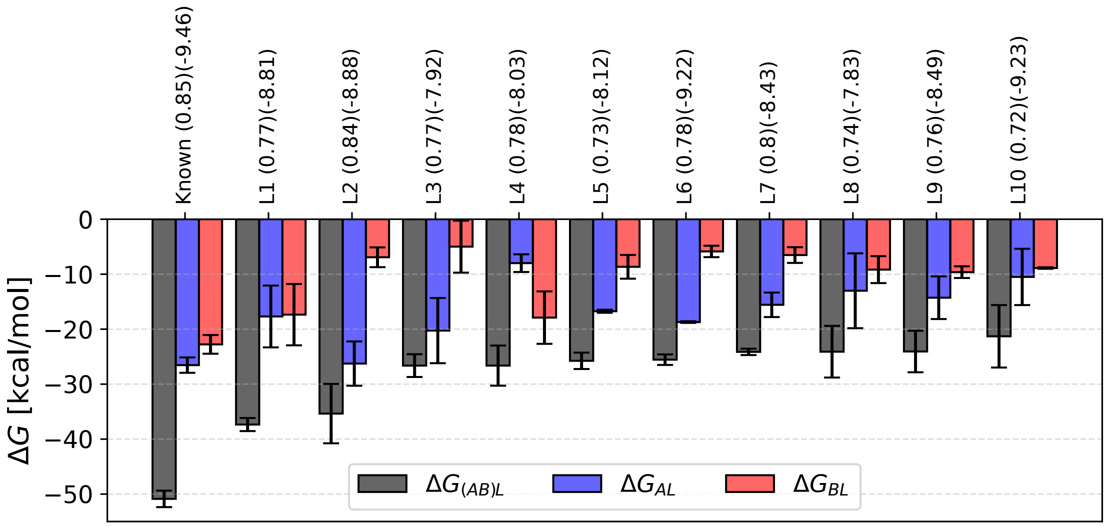

Figure S25: **MMGBSA scores for Boltz-2 binder probability filtered ligands (5ad3):** MMGBSA free energies of ligand with the protein complex (gray) and with each protein partners (light blue and light red). These ligands were selected for simulation based on their Boltz-2 binder probabilities (> 0.70). The ticks in the top depicts the Boltz-2 binder probability (lower) and Boltz-2 affinity score (upper).

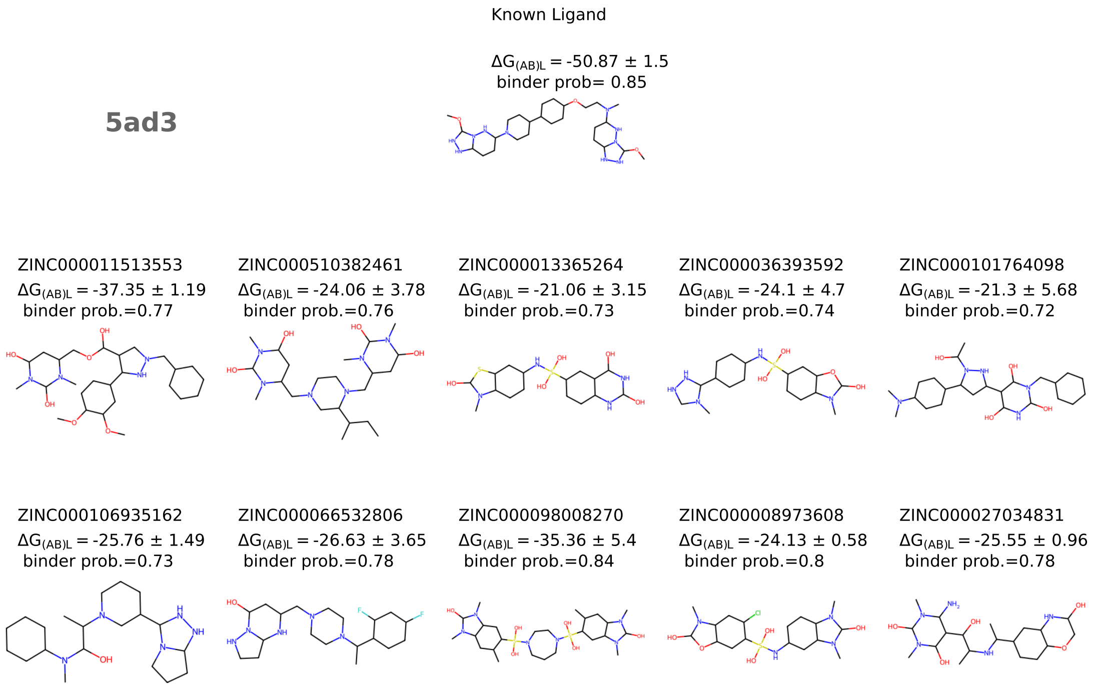

Figure S26: **Chemical structure and ZINC ID for Boltz-2 binder probability filtered ligands (5ad3):**. These ligands corresponds to the MMGBSA re-scored top ligands shown in Figure S25. The known experimental ligand is also displayed at the top. For each ligand the MMGBSA binding free energy ($\Delta G_{(AB)L}$) of the ligand with the whole protein-protein complex is shown.

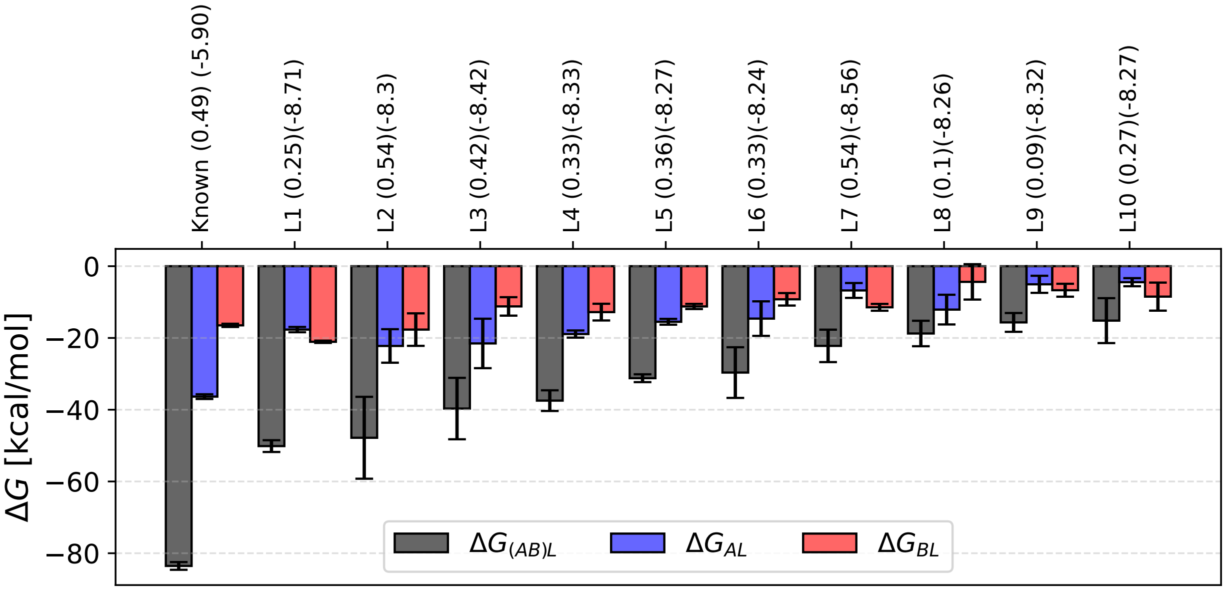

Figure S27: **MMGBSA scores for Boltz-2 binder affinity filtered ligands (4gnt):** MMGBSA free energies of ligand with the protein complex (gray) and with each protein partners (light blue and light red). These ligands were selected for simulation based on their Boltz binder affinity (< -8.1 kcal). The ticks in the top depicts the Boltz binder probability (lower) and Boltz affinity score (lower).

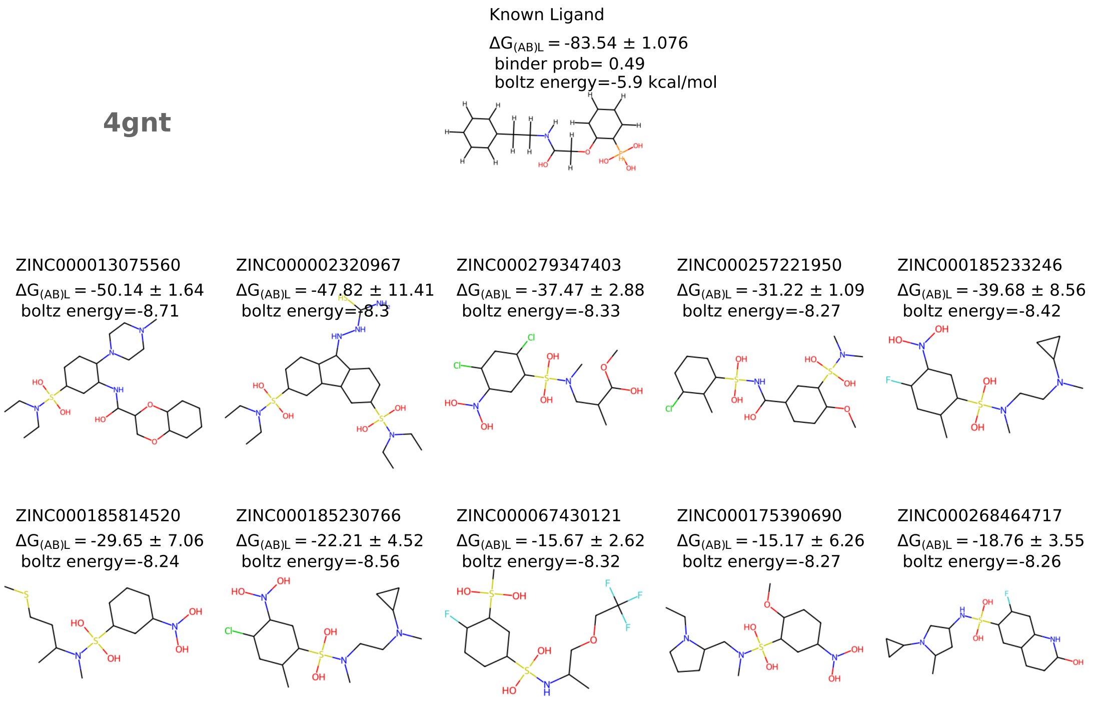

Figure S28: **Chemical structure and ZINC ID for Boltz-2 binder affinity filtered ligands (4gnt):**. These ligands corresponds to the MMGBSA re-scored top ligands shown in Figure S27. The known experimental ligand is also displayed at the top. For each ligand the MMGBSA binding free energy ($\Delta G_{(AB)L}$) of the ligand with the whole protein-protein complex is shown.

4 Finding ligands without prior pocket information: detect binding pocket using Fpocket

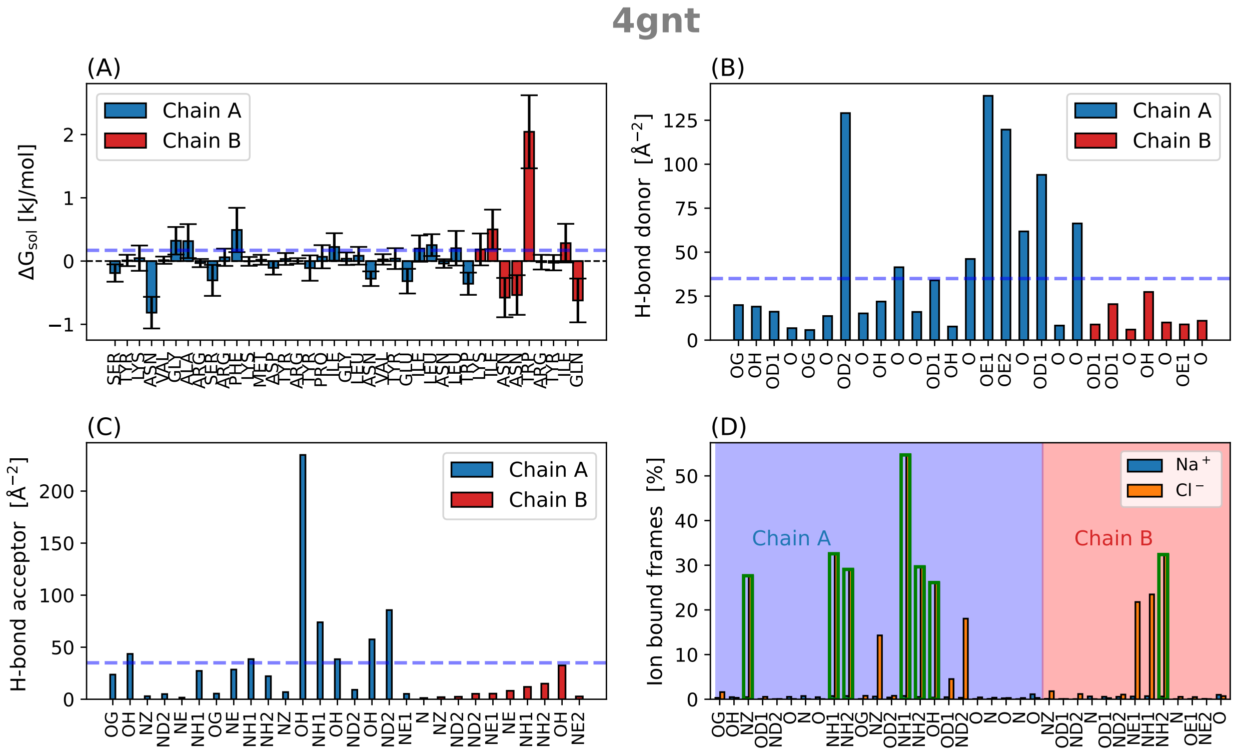

Figure S29: **Pharmacophore features from MD simulation (4gnt).** (A-D) same as in Figure S1. There are 30 features in the master pharmacophore model. Aromatic=2 (chainA=1, chainB=1), Hydrophobic=6(chainA=4, chainB=2), Hydrogen bond donor=8(chainA=8, chainB=0), Hydrogen bond acceptor=7 (chainA=7, chainB=0), Negative ion site=7 (chainA=6, chainB=1).

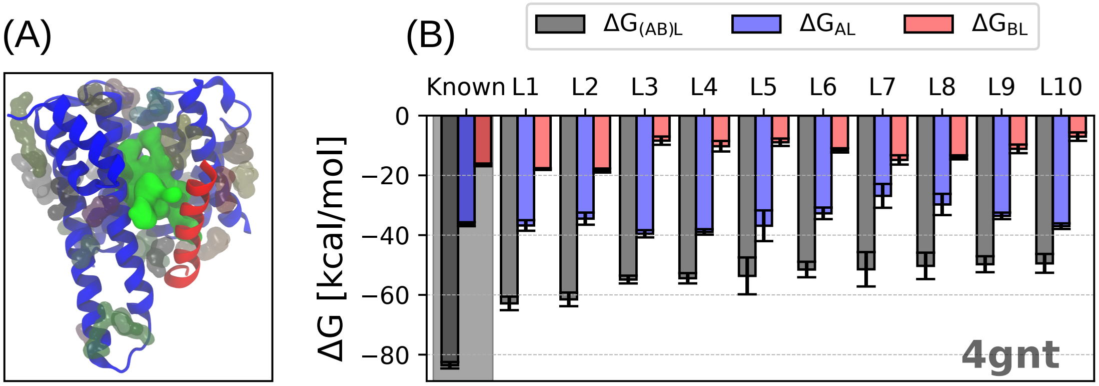

Figure S30: **Fpocket predicted pocket MMGBSA**. (A) Pockets predicted by Fpocket[2] for the 4gnt complex. The highlighted green pocket corresponds to the pocket selected by maximizing the harmonic mean of the buried surface areas of pocket probes with the two protein chains. This pocket is used for analysis of pharmacophore features. (B) MMGBSA values for known ligand and the top 10 ligands obtained from our method.

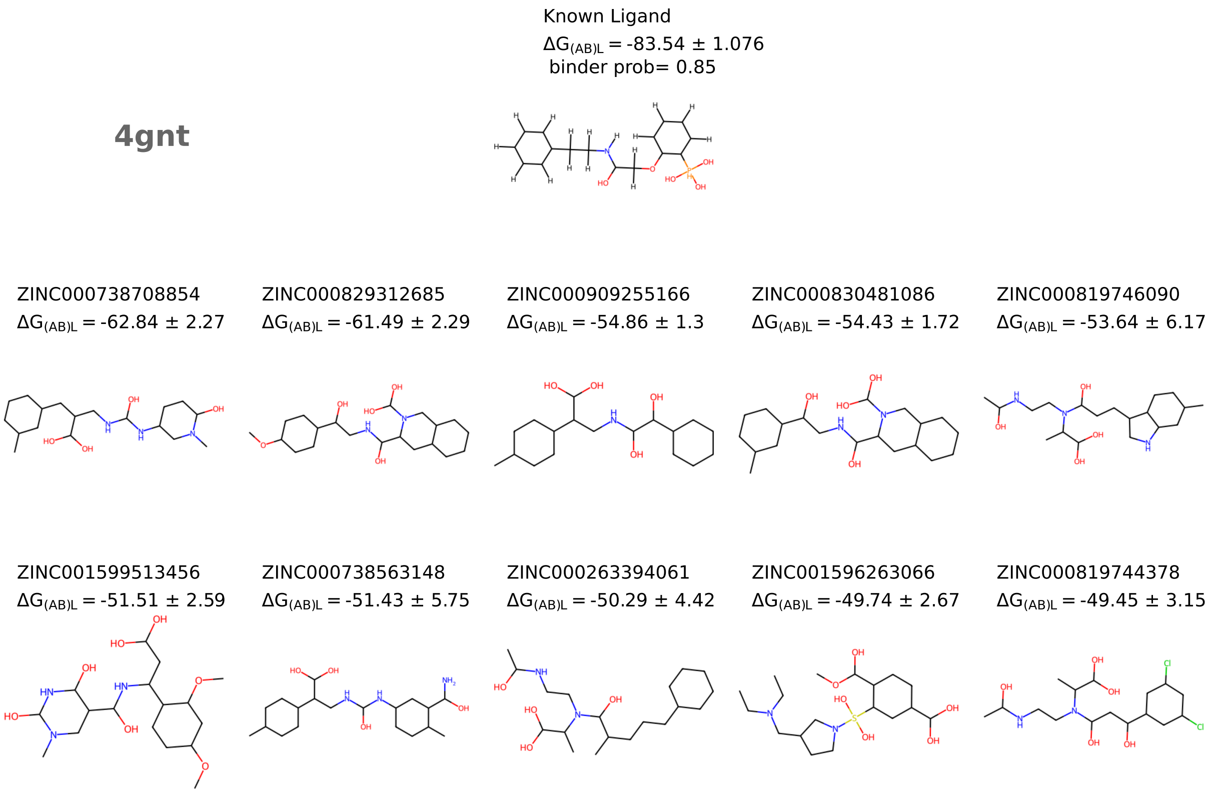

Figure S31: **Top 10 ligands for 4gnt for Fpocket predicted pocket:**. Chemical structures of and ZINC IDs of top 10 ligands displayed in Figure S30.

References

(1) Passaro, S.; Corso, G.; Wohlwend, J.; Reveiz, M.; Thaler, S.; Somnath, V. R.; Getz, N.; Portnoi, T.; Roy, J.; Stark, H.; others Boltz-2: Towards accurate and efficient binding affinity prediction. *BioRxiv* **2025**,

(2) Le Guilloux, V.; Schmidtke, P.; Tuffery, P. Fpocket: an open source platform for ligand pocket detection. *BMC Bioinform.* **2009**, *10*, 168.
